# RAC1-Dependent ORAI1 Translocation to the Leading Edge Supports Lamellipodia Formation and Directional Persistence

**DOI:** 10.1038/s41598-020-63353-5

**Published:** 2020-04-20

**Authors:** Aida M. Lopez-Guerrero, Noelia Espinosa-Bermejo, Irene Sanchez-Lopez, Thomas Macartney, Carlos Pascual-Caro, Yolanda Orantos-Aguilera, Lola Rodriguez-Ruiz, Ana B. Perez-Oliva, Victoriano Mulero, Eulalia Pozo-Guisado, Francisco Javier Martin-Romero

**Affiliations:** 1 Department of Biochemistry and Molecular Biology, School of Life Sciences and Institute of Molecular Pathology Biomarkers, University of Extremadura, Badajoz, 06006 Spain; 2 MRC- Protein Phosphorylation and Ubiquitylation Unit, School of Life Sciences, University of Dundee, Dundee, DD1 5EH Scotland United Kingdom; 3 Department of Cell Biology and Histology, University of Murcia, IMIB-Arrixaca, Murcia, 30100 Spain; 4 Department of Cell Biology, School of Medicine and Institute of Molecular Pathology Biomarkers, University of Extremadura, Badajoz, 06006 Spain

**Keywords:** Lamellipodia, Calcium signalling

## Abstract

Tumor invasion requires efficient cell migration, which is achieved by the generation of persistent and polarized lamellipodia. The generation of lamellipodia is supported by actin dynamics at the leading edge where a complex of proteins known as the WAVE regulatory complex (WRC) promotes the required assembly of actin filaments to push the front of the cell ahead. By using an U2OS osteosarcoma cell line with high metastatic potential, proven by a xenotransplant in zebrafish larvae, we have studied the role of the plasma membrane Ca^2+^ channel ORAI1 in this process. We have found that epidermal growth factor (EGF) triggered an enrichment of ORAI1 at the leading edge, where colocalized with cortactin (CTTN) and other members of the WRC, such as CYFIP1 and ARP2/3. ORAI1-CTTN co-precipitation was sensitive to the inhibition of the small GTPase RAC1, an upstream activator of the WRC. RAC1 potentiated ORAI1 translocation to the leading edge, increasing the availability of surface ORAI1 and increasing the plasma membrane ruffling. The role of ORAI1 at the leading edge was studied in genetically engineered U2OS cells lacking ORAI1 expression that helped us to prove the key role of this Ca^2+^ channel on lamellipodia formation, lamellipodial persistence, and cell directness, which are required for tumor cell invasiveness *in vivo*.

## Introduction

To be efficient, cell migration requires tight spatiotemporal control of cellular signaling. Together with other molecules involved in the control of cell migration, calcium ion (Ca^2+^) has emerged as a key modulator of cytoskeleton reorganization in migrating cells^1^. Ca^2+^ signaling regulates cell migration in different aspects: the turnover of focal adhesions^2,3^, the contraction of actomyosin fibers (reviewed in^4^), plasma membrane ruffling^5,6^, and the formation of podosomes^7^. Indeed, in migrating cells a gradient is observed in the concentration of free cytosolic Ca^2+^ ([Ca^2+^]_i_) so that on the front side the [Ca^2+^]_i_ is slightly lower than on the rear part of the cell^8,9^. This gradient of Ca^2+^ causes the Ca^2+^-dependent signaling to be polarized, as indeed is polarized the cytoskeleton in migrating cells. This difference in the [Ca^2+^]_i_ is due to a differential subcellular localization of Ca^2+^ channels and pumps along the front-rear axis. For example, at the leading edge there is a greater concentration of PMCA (plasma membrane Ca^2+^-ATPase) and a higher density of SERCA (sarco(endo)plasmic reticulum Ca^2+^-ATPase), which facilitates a localized decrease of [Ca^2+^]_i_^10^. At the rear part of the migrating cell, the trailing edge, a wide range of Ca^2+^ channels increase [Ca^2+^]_i_ and facilitate the activation of myosin light-chain kinase (MLCK) and Rho kinase-dependent processes which are necessary to stimulate actomyosin contraction and facilitate cell retraction^11^. For these reasons, the subcellular localization of the different plasma membrane Ca^2+^ channels is critical when performing their function as cell migration regulators, either at the leading edge, the trailing edge, or modulating focal adhesion turnover.

The influx of Ca^2+^ through store-operated Ca^2+^ channels (SOC channels) regulates the migration of both cancer and non-cancer cells^12–17^. This Ca^2+^ influx pathway is controlled by a number of plasma membrane Ca^2+^ channels, including the ORAI protein family and some members of the TRPC protein family (reviewed in^18^). Amongst these SOC channels, much attention has focused on ORAI1 since upregulation of its expression has been reported in a variety of cancer cell lines and primary tumors^19,20^, pointing to ORAI1 as being a potential target for cancer therapy. ORAI1 is involved in the epithelial to mesenchymal transition of breast cancer cells^21^, and the positive regulation of cell migration, but not in proliferation^12^. The closely related homolog ORAI3, however, is involved in the regulation of proliferation and cell death suppression of cancer cells^19,22,23^, but plays a minor role in cell migration. The control of the subcellular localization of ORAI1 is largely unknown, and its study should help explain the differential roles of ORAI homologs.

In this regard, previous work has shown that ORAI1 presents a subcellular localization that overlaps with cortactin (CTTN) in HeLa cells, U2OS cells, and myoblasts^5^. CTTN is a well-known regulator of lamellipodia formation^24^, and therefore is a marker of the leading edge. The polarized localization of ORAI1 at the leading edge constitutes a good match with the polarized localization of phospho-STIM1^5^, a pool of STIM1 that is phosphorylated by the ERK1/2 kinase. The function of STIM1 is to act as a Ca^2+^ sensor within the intraluminal side of the endoplasmic reticulum (ER) and to activate the opening of ORAI1 upon partial depletion of Ca^2+^ concentration within the ER^25^. In this way, i.e., being STIM1-dependent, ORAI1 can act as a SOC channel, although this channel can also be activated by Ca^2+^ store-independent mechanisms^26,27^.

The polarized localization of STIM1 had been reported in a work that had shown an increasing gradient of diacylglycerol towards the front that promotes migration of endothelial cells^10^. To reach the leading edge, STIM1 requires the binding to microtubules through the microtubule plus-end binding protein EB1^10^. However, the leading edge of migrating cells is devoid of microtubules, and the formation of filopodia and lamellipodia is driven by actin^28^, so an additional explanation for this re-localization was required. The explanation proposed was a mechanism of STIM1 regulation by phosphorylation^29,30^. The leading edge is enriched in receptor tyrosine kinases (RTKs)^31^, and many of these RTKs activate the ERK1/2 kinase pathway. ERK1/2 phosphorylates STIM1 at residues Ser575, Ser608, and Ser621^29,32^ which are close to the sequence that directly binds to EB1, leading to the dissociation of STIM1 from EB1^29^, and enabling the dissociation of STIM1 from microtubules in the microtubule-free lamellipodia. However, the mechanisms that regulate the localization of ORAI1 at the leading edge remain far from clear.

The small GTPase RAC1 regulates the localization of CTTN and the WAVE regulatory complex (WRC)^33–35^. RAC1 is a downstream effector of RTKs, and is highly enriched at the leading edge^36^, making this small GTPase a candidate for the regulation of the subcellular localization of ORAI1. We used an osteosarcoma cell line with high metastatic potential, proven by a xenotransplant in zebrafish larvae, to analyze ORAI1 enrichment at the leading edge by means of immunofluorescence co-localization, and co-precipitation with cortactin, ARP2/3, and CYFIP1, which are at highly enriched levels in lamellipodia. We found that RAC1 potentiated ORAI1 translocation to the leading edge, and that this was sensitive to RAC1 inhibition, either by a chemical inhibitor or by a dominant negative mutation in RAC1. Our results demonstrate that RAC1 controls ORAI1 localization at the leading edge where this channel regulates lamellipodia formation, lamellipodial persistence, and cell directness, which are required for efficient cell migration and tumor cell invasiveness *in vivo*.

## Results

### ORAI1 potentiates lamellipodia formation and directional persistence

**Figure 1:**
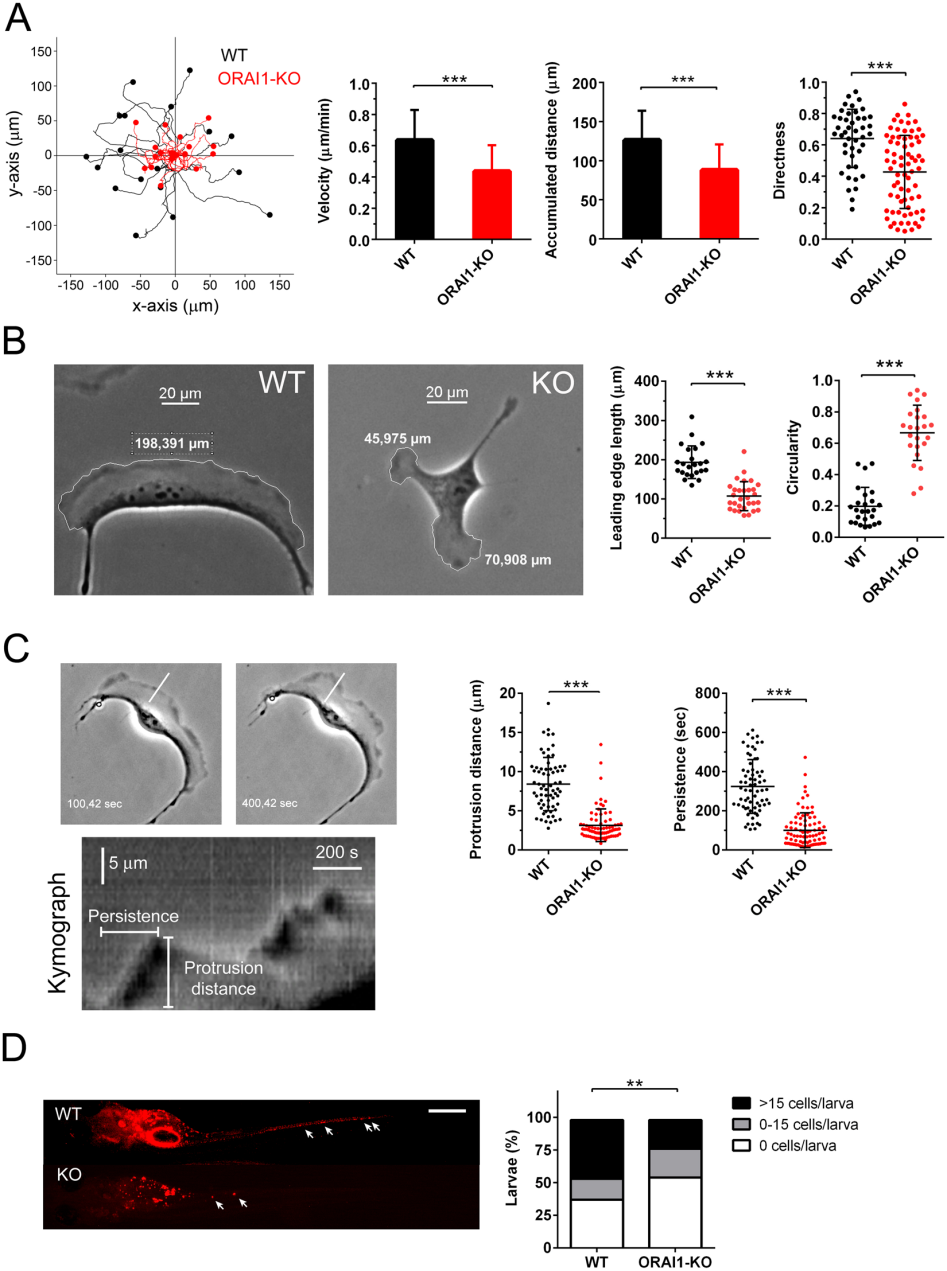
Genetic ablation of ORAI1 reduced motility, directness, lamellipodia formation, and invasion of U2OS cells. *Panel A*: Wild-type U2OS cells (black symbols) and ORAI1-KO cells (red symbols) were monitored for speed, accumulated distance, and directness in a 2D random motility assay (total assay time = 200 min). Representative traces of 19 cells/condition are shown in the left figure. Data from 3 independent assays/condition (n = 44 WT cells; n = 78 KO cells) are shown as bar charts or dot plot. *Panel B*: The leading edge length was measured using bright-field images of wild-type cells and ORAI1-KO cells. Data from 3 independent experiments (>22 cells/condition) were measured. Bar = 20 μm. Circularity index was measured from 2 independent experiments (>20 cells/condition). *Panel C*: Protrusion distance and persistence were measured from 2D random motility assays. Using a 3-pixel width line drawn at the leading edge, protrusion distance and persistence were measured from the resulting kymograph (left panels). An example of a wild-type cell at two different times and the resulting kymograph are shown. Plotted data are individual data from 2 independent experiments. *Panel D*: The experimental design of xenotransplants of wild-type U2OS and ORAI1-deficient cells in casper zebrafish larvae is represented in the Supplementary Fig. S5. Imaging to analyze U2OS cells invasion was performed at 5 days post injection (dpi). Left panels: Representative images of wild-type and ORAI1-KO U2OS cells dissemination in zebrafish at 5 dpi. Magnification bar: 500 µm. Right panel: Percentage of invaded larvae of both genotypes with different invasion levels. Data shown in bar chart are from 2 independent experiments (n = 119 larvae injected with WT cells, n = 87 larvae injected with ORAI1-KO cells). **p < 0.01 according to Chi-square tests.

As noted above, we have recently reported that the plasma membrane Ca^2+^ channel ORAI1 is enriched at the leading edge of migrating cells, where it colocalizes with CTTN^5^. In those specific areas, ORAI1 and its regulator STIM1 are critical mediators for the Ca^2+^ entry required to sustain membrane ruffling. To further evaluate the role of ORAI1 at the leading edge, we analyzed different features of random cell migration on collagen-coated plates. Using U2OS cells edited by CRISPR/Cas to knock-out ORAI1 gene expression^5^, we observed that cell migration speed and total distance were greatly inhibited by the ORAI1 deficiency (Fig. 1A, and Supplementary movie S1 and S2). More important was the fact that cell directness was significantly reduced (from 0.63 ± 0.03 to 0.43 ± 0.03) (Fig. 1A). As a control, ORAI1-KO cells were transduced with retrovirus for the stable expression of ORAI1 (Supplementary Fig. S3), and this experiment proved that the over-expression of ORAI1 and the consequent rescue of ORAI1-dependent Ca^2+^ entry, reversed the inhibitory effect on cell directness observed in the ORAI1-KO cell line leading to the conclusion that the impairment of cell polarity was specifically due to the absence of ORAI1.

The analysis of the leading edge length, i.e., the length of the sheet extending along the front of the moving cells, revealed that ORAI1 also plays a role in the formation or the stabilization of lamellipodia because ORAI1-deficient cells had smaller lamellipodia than wild-type cells (Fig. 1B and Supplementary Fig. S4). Indeed, the analysis of the cells shows the ORAI1-KO cells to be weakly polarized (Fig. 1B and Supplementary Fig. S4). The combination of decreased polarity (or increased circularity) and shortened lamellipodia led us to analyze protrusion distance and lamellipodial persistence because a smaller lamellipodia could be the result of low levels of persistence or distance covered by the nascent lamellipodia. Our analysis confirmed that removal of ORAI1 triggered a significant reduction in both the extension of newly formed lamellipodia and in lamellipodial persistence (Fig. 1C), suggesting that there are defects in the formation of lamellipodia in the absence of ORAI1.

Directional persistence and polarization are two key factors for successful cell migration and invasion *in vivo*. For this reason, we analyzed invasion rates in an *in vivo* model using xenotransplants in zebrafish larvae. Casper zebrafish larvae were micro-injected with wild-type or ORAI1-KO U2OS cells, and 5 days post-injection the larvae were analyzed for cell dissemination by fluorescence microscopy (see experimental design in Supplementary Fig. S5). The results showed a higher level of tumor cells in the larvae when wild-type U2OS cells were injected (Fig. 1D). The deficiency in ORAI1 significantly reduced the dissemination of osteosarcoma U2OS cells, a finding that we propose is directly linked to the reduction in cell migration rate, in directional persistence, and in protrusion formation.

### EGF triggers the association between ORAI1 and CTTN

**Figure 2:**
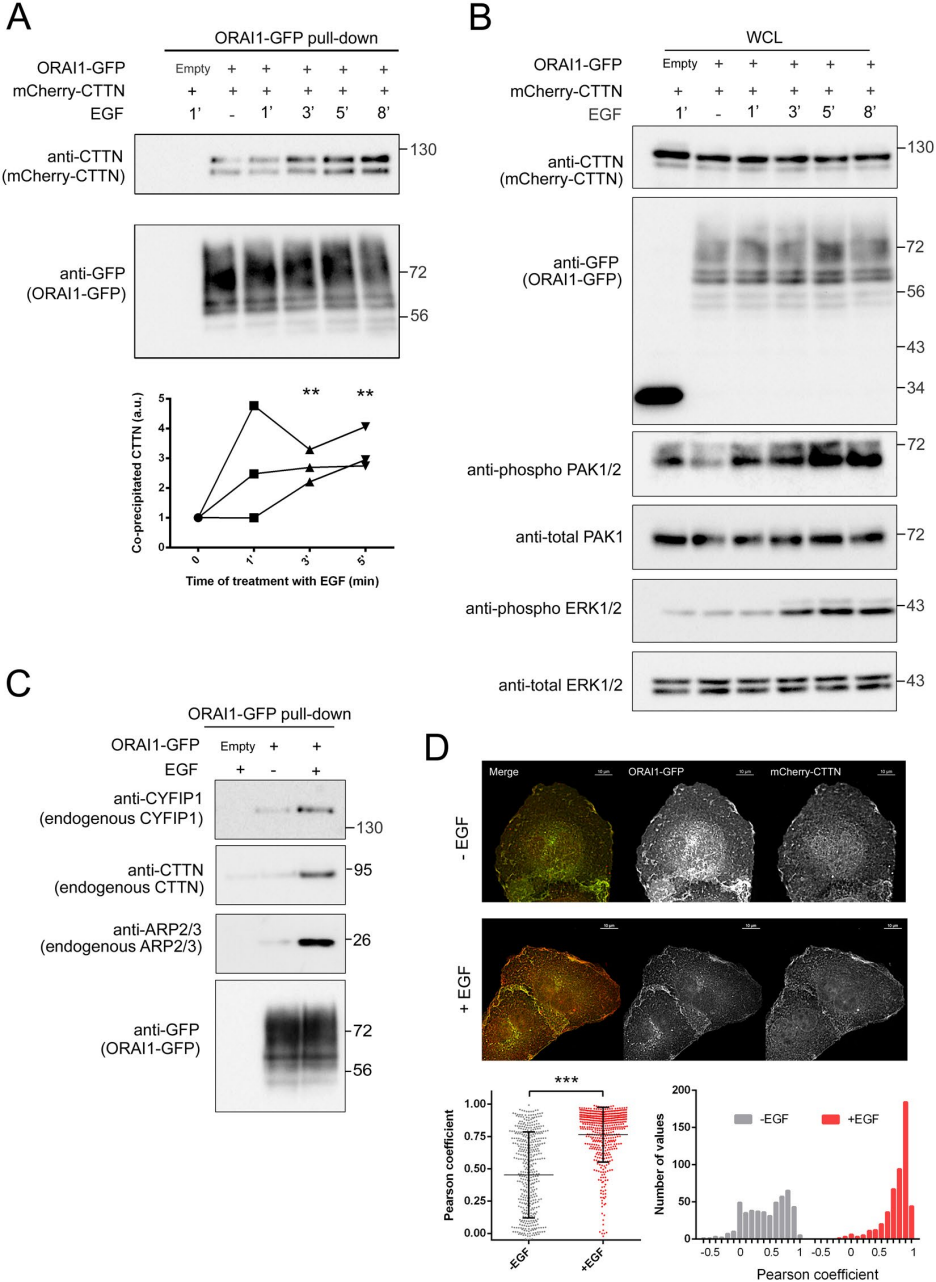
EGF potentiated ORAI1 binding to CTTN, CYFIP1, and ARP2/3. *Panel A*: U2OS cells transfected for the expression of ORAI1-GFP (or the empty vector, i.e., GFP only) and mCherry-CTTN were starved overnight with FBS-free medium and treated with 50 ng/ml EGF for the times indicated in the figure. Images are representative of 3 independent experiments. From total lysates, ORAI1-GFP was pulled down and the co-precipitated mCherry-CTTN analyzed by immunoblot. In co-IP assays, proteins were separated using 6.5% acrylamide gels. Total ORAI1-GFP pulled down was assessed with an anti-GFP antibody. The quantification of ORAI1-CTTN co-precipitation was evaluated with an anti-CTTN antibody from 3 independent experiments (scatter plot). *Panel B*: Whole cell lysates (WCL) from *panel A* were subjected to electrophoresis on 10% acrylamide gels, blotted, and assessed for the level of mCherry-CTTN, ORAI1-GFP, phospho-PAK1/2, total-PAK1, phospho-ERK1/2, and total-ERK1/2. *Panel C*: U2OS cells were transfected for the expression of ORAI1-GFP or the empty vector. Cells were starved overnight with FBS-free medium and treated with 50 ng/ml EGF for 3 min. ORAI1-GFP was pulled down, and the co-precipitated endogenous CYFIP1, CTTN, and ARP2/3 were analyzed by immunoblot. Total ORAI1-GFP pulled down was assessed with an anti-GFP antibody. Blots are representative of 3 independent experiments. Full-length blots are presented in Supplementary Fig. S13. *Panel D*: U2OS cells transfected for the expression of ORAI1-GFP and mCherry-CTTN were starved overnight with FBS-free medium and treated with 50 ng/ml EGF for 3 min (+EGF). Controls without the addition of EGF (-EGF) were processed in parallel. Fixed cells were analyzed under epifluorescence microscopy to evaluate the Pearson correlation coefficient of GFP and mCherry in the cell periphery only. Circular ROIs of 1.1–1.18 μm^2^ were set over the cell periphery that remained free of cell-cell contacts. Pearson correlation coefficient values within the range 0–1 are plotted in the bottom left panel. Right panel shows the histogram of the frequency distribution of Pearson coefficient values.

Because EGF modulates cell migration and motility in epithelial cells and EGF receptors are enriched at the leading edge^31^, we investigated the binding of ORAI1 to CTTN in U2OS cells stimulated with EGF as an strategy to study the possible translocation or re-localization of ORAI1 to the leading edge in response to EGF. Cells were starved in FBS-free RPMI 1640 medium without phenol red for 8–10 h and then stimulated with 50 ng/ml EGF in the same medium. ORAI1-CTTN binding was monitored by ORAI1-GFP pulldown and subsequent analysis of co-precipitated mCherry-CTTN (Fig. 2A). The time course of EGF stimulation was evaluated by monitoring the levels of (i) phospho-PAK1/2 (residues Thr423/Thr402), a well characterized serine-threonine kinase activated by the small GTPase RAC1 and a downstream mediator of EGFR, and (ii) phospho-ERK1/2, since the MAPK pathway becomes activated by EGF (Fig. 2B). The increase in PAK1/2 and ERK1/2 phosphorylation was observed after 1–3 min of stimulation with EGF. Within this time window, we analyzed the co-precipitation between ORAI1 and CTTN, observing greater binding after stimulation, and this increase reached statistical significance after 3 min of treatment with EGF (Fig. 2A). Because CTTN is a molecular marker of lamellipodia, this result suggests that EGF triggers the recruitment of ORAI1 to the leading edge. Also, when U2OS cells were stimulated with EGF under the above conditions, ORAI1-GFP was observed to co-precipitate with both endogenous CTTN and with endogenous CYFIP1 (cytosolic FMR-interacting protein 1) (Fig. 2C), also known as SRA-1 (specifically RAC1-associated protein 1)^37^, one of the subunits of the WRC, a molecular complex enriched at the leading edge.

CTTN plays a key role in lamellipodia. It binds to the ARP2/3 complex in existing actin fibers (F-actin) to facilitate the formation of actin fiber branches. We therefore also monitored the co-precipitation of ORAI1 with the endogenous ARP2/3 complex (Fig. 2C). This analysis confirmed the recruitment of ORAI1 to peripheral areas enriched in CTTN and ARP2/3, i.e., the translocation of ORAI1 to the leading edge where CTTN and ARP2/3 are cooperating to form lamellipodia.

These results were confirmed by the use of epifluorescence microscopy to monitor ORAI1-GFP and mCherry-CTTN colocalization before and after the treatment with 50 ng/ml EGF (Fig. 2D). Pearson correlation coefficient was calculated to quantify the degree of co-localization in both conditions. For this quantitation we analyzed the cell periphery that remained free of cell-cell contacts. The results indicated that there was a significant enrichment of ORAI1-CTTN co-localization at the cell periphery in response to the stimulation with EGF.

### RAC1 activity mediates the translocation of ORAI1 to the leading edge

An upstream regulator of CTTN, which is activated by EGF, is the member of the Rho family of GTPases RAC1^34^. It is also known that RAC1 is enriched at the leading edge of migrating cells^36^ where it stimulates the recruitment of CTTN to branched actin networks^33,34^. In this regard, RAC1 also co-precipitates with the WAVE regulatory complex, a protein complex that regulates lamellipodia formation (reviewed in^38^), although it is not part of the core complex.

To investigate the role of RAC1 in the control of ORAI1 subcellular localization, we first monitored RAC1 localization in cell lines expressing EGFP-RAC1 wild-type or EGFP-RAC1^Q61L ^(a constitutively active form of RAC1) in FBS-containing assay medium. This mutation impairs GTPase activity which means that RAC1 is locked in the GTP-bound conformation^39^.

**Figure 3:**
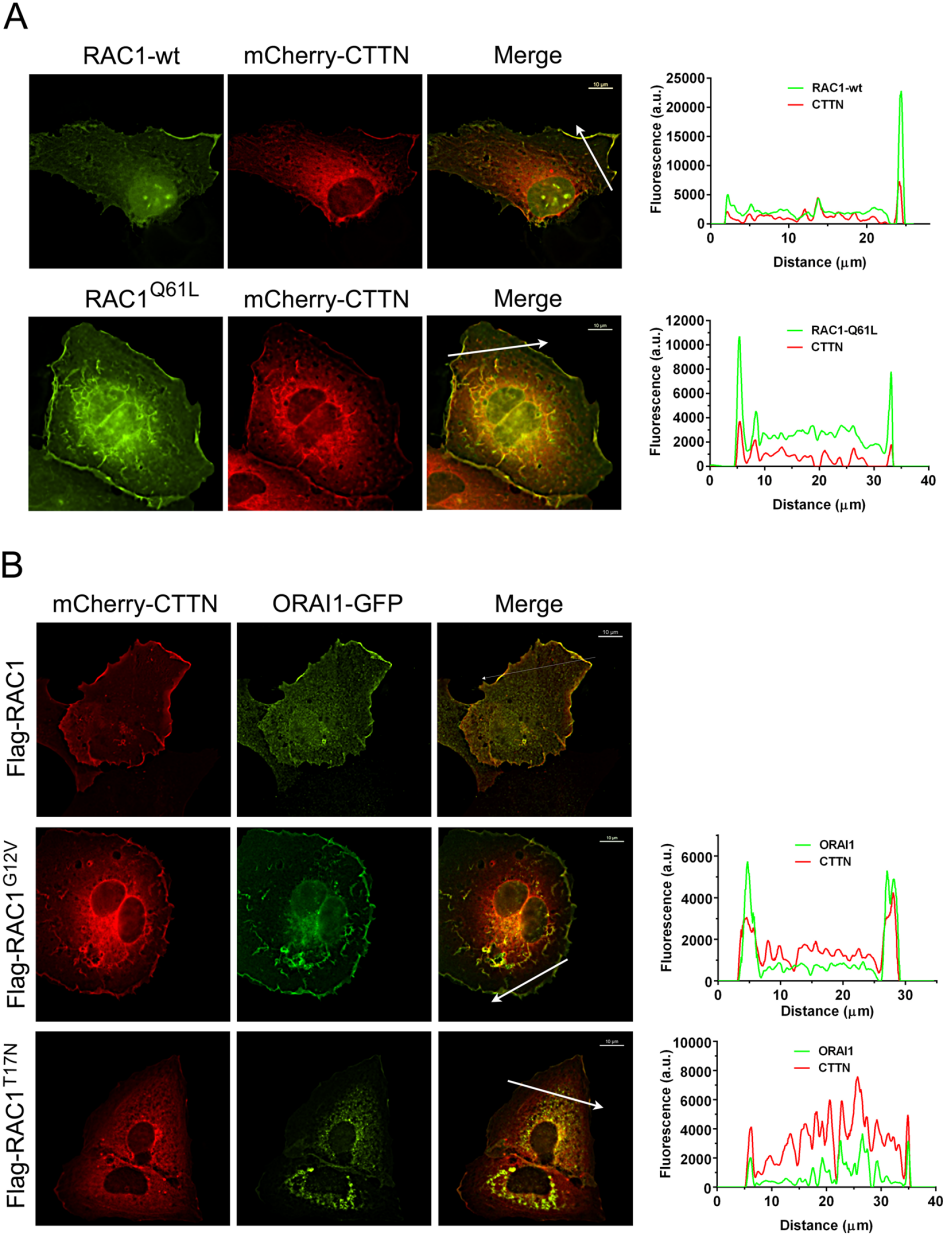
Activation of RAC1 triggered the translocation of ORAI1 to CTTN-containing cell periphery. *Panel A*: Cells growing onto collagen-coated coverslips in FBS-containing DMEM were transfected for the expression of mCherry-CTTN and EGFP-RAC1 (wt) or EGFP-RAC1^Q61L^ (active RAC1). At 36 h after transfection, cells were fixed and visualized under wide-field epifluorescence microscopy. Cherry and GFP channels were recorded sequentially using independent filter blocks. The two images were merged, and the fluorescence of both channels was measured over the arrow depicted in the image. Fluorescence values, plotted on right panels, were quantified with the NIS-Elements AR software. Images are representative of a minimum of 32 cells per condition from 3 independent experiments. Bar = 10 μm*. Panel B*: Cells stably expressing Flag-RAC1 (wild-type), Flag-RAC1^G12V^, or Flag-RAC1^T17N^ were transfected for the expression of mCherry-CTTN and ORAI1-GFP. As in panel A, cells were analyzed under epifluorescence microscopy, and Cherry and GFP channels were recorded. Images are representative of >26 cells per condition from 3 independent experiments. Bar = 10 μm.

Consistent with the role of RAC1 as an upstream regulator that triggers the translocation of CTTN to the cell periphery, we observed that the constitutively active mutant RAC1^Q61L^ promoted a peripheral localization of CTTN (Fig. 3A). The line scan of the fluorescence signal showed there to be significant enrichment of both RAC1 and CTTN at the cell periphery under each of the two conditions. We must emphasize, however, that, in contrast to what we observed with RAC1 wild-type, there was no polarization in the CTTN localization when RAC1 was permanently active (RAC1^Q61L^). This finding can be easily explained by the fact that RAC1^Q61L^ activity is independent of polarized receptor tyrosine kinases and is able to trigger lamellipodia formation over the entire cell periphery.

We then monitored ORAI1 and CTTN localization in cell lines stably expressing (i) Flag-RAC1 wild-type, (ii) Flag-RAC1^G12V^ (another constitutively active form of RAC1 that impairs the GTPase activity leading to the locking of RAC1 in the GTP-bound conformation), and (iii) Flag-RAC1^T17N^ (a dominant negative mutant of RAC1) (Fig. 3B). As well as the Q61L, all these mutant forms of RAC1 have been widely used in other cell types to study the role of RAC1 in membrane ruffling, lamellipodia formation, and cell invasion^40–43^. In Flag-RAC1 (wt)-overexpressing cells, ORAI1-GFP was found polarized in restricted areas of CTTN. However, the active form of RAC1^G12V^ triggered significant re-localization of ORAI1 to the entire cell periphery, with an identical localization profile to that found for CTTN in these conditions. Similar localization of ORAI1 was found when the cells were transfected for the expression of mCherry-RAC1^G12V ^and ORAI1-GFP (Supplementary Fig. S6). In contrast, the dominant negative mutant of RAC1, RAC1^T17N^, reduced the translocation of CTTN to the periphery in the same way as it inhibited the peripheral localization of ORAI1.

To quantify the extension of ORAI1 enrichment at CTTN-areas, we assessed the level of cortical ORAI1-GFP/total ORAI1-GFP in individual cells and the ratio cortical CTTN/total CTTN. The analysis revealed that RAC1^G12V^ triggered a significant increase of cortical ORAI1 and CTTN, whereas RAC1^T17N^ reduced the level of both proteins at the cell cortex. The relative level of cortical ORAI1/CTTN did not change between these experimental conditions (Supplementary Fig. S7). These results suggest that both proteins have a similar behavior when RAC1 is modulated by the mutations used in the study.

**Figure 4:**
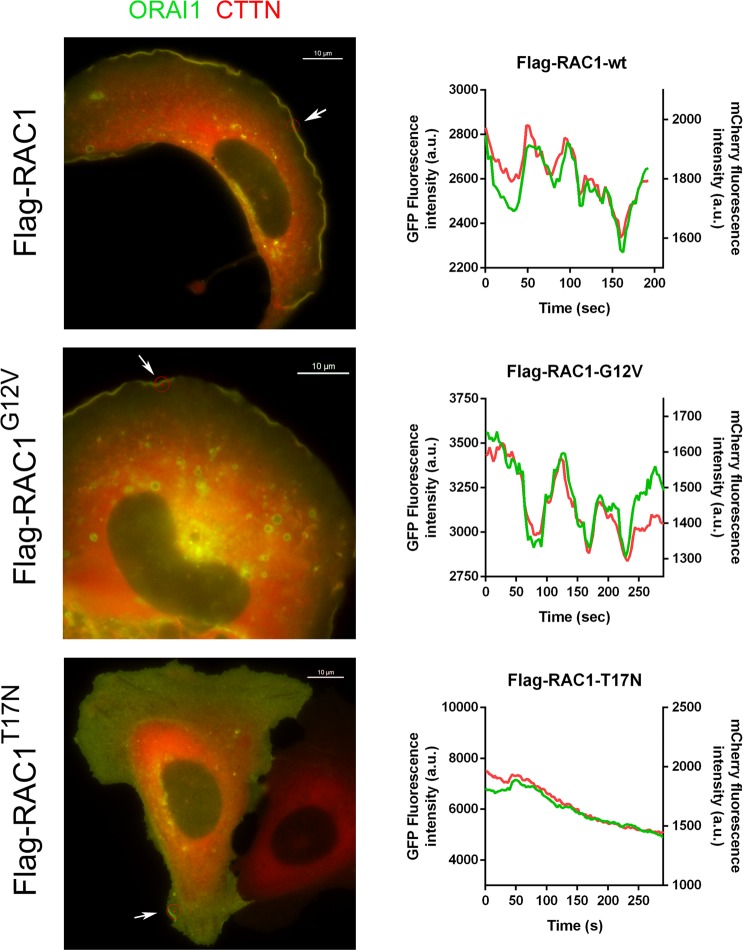
RAC1 regulated the dynamics of peripheral ORAI1-CTTN and ruffling. U2OS cells stably expressing Flag-RAC1 (wild-type), Flag-RAC1^G12V^ or Flag-RAC1^T17N^, were transfected for the transient expression of ORAI1-GFP and mCherry-CTTN. Fluorescence was monitored in live cells in Leibovitz’s L-15 medium supplemented with 10% FBS. The ROI indicated by the arrow was selected to assess the variation of fluorescence intensity (right panels). Emission of fluorescence of ORAI1-GFP (green line) and mCherry-CTTN (red line) was recorded every 3 sec for 4 min. Full time-lapse sequences are shown as Supplementary Movies S8 (for wild-type RAC1), S9 (RAC1^G12V^), S10 (RAC1^T17N^). Bar = 10 μm.

In addition to the localization of ORAI1, we studied the ruffling of the leading edge in response to the activation or inhibition of RAC1. Cells stably expressing Flag-RAC1 wild-type, Flag-RAC1^G12V^, and Flag-RAC1^T17N^ were transfected for the transient expression of ORAI1-GFP and mCherry-CTTN. Cells growing in FBS-containing medium were visualized under epifluorescence microscopy and the cortical ruffling was evaluated as described previously by our group^5^. Supplementary Movies S8, S9, and S10 show time-lapse sequences with the dynamics of GFP/Cherry in Flag-RAC1 wild-type, Flag-RAC1^G12V^, and Flag-RAC1^T17N^, respectively. Figure 4 depicts a single frame from the time-lapse experiments. The analysis of the fluorescence at the cell cortex shows the spiking of the fluorescence for both tags, which was strongly inhibited in Flag-RAC1^T17N^-expressing cells.

**Figure 5:**
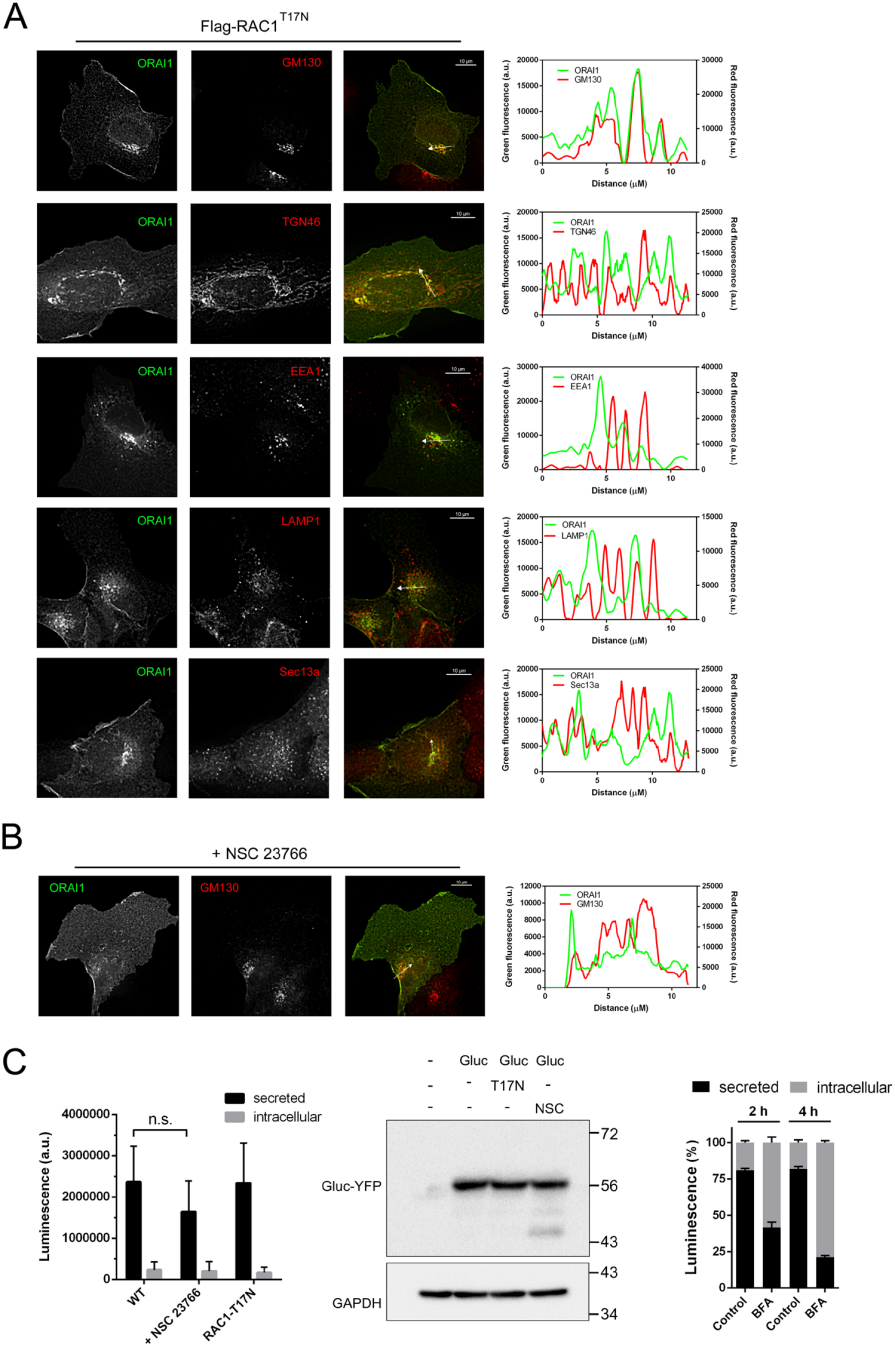
RAC1^T17N^ blocked the transport of ORAI1 to the cell surface. *Panel A*: U2OS cells stably expressing Flag-RAC1^T17N^ were transfected for the transient expression of ORAI1-GFP. Cells were fixed 24 h after transfection, and used for the immunolocalization of GM130, TGN46, EEA1, LAMP1, and Sec13a. Secondary antibodies were labelled with Alexa Fluor 594. Images are representative of 2 independent experiments (>20 cells per condition). Fluorescence of both channels was measured over the arrow depicted in the image, which was placed over the intracellularly trapped GFP- signal. *Panel B*: U2OS cells were treated with 50 μM NSC 23766 for 8 h, fixed, and the immunolocalization of GM130 was performed as in *panel A*. Images are representative of 26 cells from 2 independent experiments. Bar = 10 μm. *Panel C: Left*: Cells were cultured on 96-well plates in DMEM + 10% FBS medium. The secreted and the intracellular luciferase activity were measured after 28 h of culture. The treatment with 50 μM NSC 23766 was performed during the last 8 h of culture. The overexpression of Flag-RAC1^T17N^ was triggered with doxycycline during the last 22 h of culture. Data from n = 15 wells and 3 independent experiments are shown as bar chart. *Middle*: Intracellular Gluc-YFP protein was evaluated from cell lysates by immunoblot using an anti-GFP antibody. *Right*: Cells were treated with 5 μg/ml brefeldin A for 2–4 h to assess the inhibition of the secretory pathway, as a control of the experiment.

In addition to the translocation of ORAI1 to lamellipodia, also striking was the alteration of the intracellular/peripheral ratio of ORAI1 stimulated by the activation of RAC1. As observed in cells expressing RAC1^T17N^, the inhibition of RAC1 activity triggered accumulation of ORAI1 in intracellular vesicles compared with RAC1 (wt)-expressing cells (Fig. 3B). We have investigated the intracellular trapped ORAI1 using markers for ER-Golgi transport (Sec13a), cis-Golgi (GM130), trans-Golgi (TGN46), endosomes (EEA1), and lysosomes (LAMP1), and found that these vesicles were GM130 positive (Fig. 5). Therefore, an important conclusion from this experiment is the partial inhibition of ORAI1 translocation to the plasma membrane due to RAC1 inactivation. Because NSC 23766 is a widely used inhibitor of RAC1 we also investigated whether the treatment of cells with NSC 23766 led to ORAI1 accumulation into cis-Golgi vesicles. In this latter case, ORAI1 was found much more diffusively distributed throughout the cytoplasm, without evident accumulation at Golgi (Fig. 5B), suggesting that the pharmacological inhibition of RAC1 did not impaired the traffic between ER and Golgi.

To investigate whether RAC1 inhibition was impairing the entire secretory pathway we generated a stable cell line expressing the *Gaussia* luciferase, as described previously^44^. Then, we measured the secreted luciferase activity as a readout of the secretory pathway status, and we found that luciferase secretion was not inhibited by the overexpression of Flag-RAC1^T17N^ (Fig. 5C) nor by the treatment of cells with NSC 23766, validating the use of this inhibitor in subsequent experiments. As a control of the experiment, we used brefeldin A, a well-known inhibitor of the ER-Golgi transport that inhibited the secretion of the *Gaussia* luciferase.

### RAC1 inhibition reduced ORAI1 translocation and impaired cell migration

**Figure 6:**
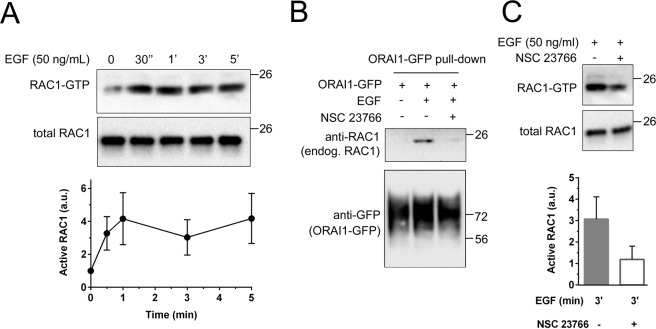
RAC1 co-precipitated with ORAI1 upon stimulation with EGF. *Panel A:* Cells were starved overnight in FBS-free cultured medium and then treated with 50 ng/ml EGF. At the indicated times, endogenous RAC1 activation (GTP-bound RAC1) was analyzed with a pull-down assay to evaluate the amount of RAC1 able to bind PAK1-PBD (see Methods section). Total RAC1 was analyzed as a loading control of the immunoblot. The blot is representative of 3 independent experiments and the quantification of active RAC1 was performed by analyzing the 3 independent experiments. *Panel B*: Cells were transfected for the expression of ORAI1-GFP, starved overnight with FBS-free medium and treated with 50 ng/ml EGF for 3 min. When required, cells were pre-incubated with 50 μM NSC 23766 for 8 h before the treatment with EGF. Then, ORAI1-GFP was pulled down with GFP beads, and the level of endogenous RAC1 co-precipitated was analyzed by immunoblot. The blot is representative of 3 independent experiments. *Panel C*: Cells were starved overnight in FBS-free cultured medium, treated with NSC 23766 when required, and then treated with 50 ng/ml EGF for 3 min. Inhibition of RAC1 by NSC 23766 was assessed with a pull-down assay to evaluate active RAC1, as in panel A. The blot is representative of 3 independent experiments. Quantification of RAC1 inhibition by NSC 23766 was performed by analyzing the 3 independent experiments. GTP-bound RAC1 levels were normalized with values obtained in the absence of stimulation (time = 0 min). Full-length blots are presented in Supplementary Fig. S13.

To investigate further the role of RAC1 in the localization of ORAI1, FBS-starved cells were stimulated with EGF, and RAC1 activity in these experimental conditions was evaluated by a classical pull-down with GST-PAK1 protein-binding domain (PBD) and the subsequent analysis of co-precipitated RAC1 (Fig. 6A). The results demonstrated that RAC1 became activated within the first 30 sec-1 min of treatment with EGF, i.e., slightly earlier than the co-precipitation of ORAI1 with CTTN, ARP2/3, and CYFIP1 (see Fig. 2), in agreement with an upstream activation of RAC1 when compared with the effect observed in ORAI1-CTTN co-precipitation. Moreover, endogenous RAC1 co-precipitated with ORAI1-GFP in response to EGF (Fig. 6B), and the RAC1 inhibitor NSC 23766 inhibited the RAC1-ORAI1 co-precipitation observed upon stimulation with EGF. This result indicated that ORAI1-GFP binds to a molecular complex containing active RAC1. The efficiency of NSC 23766, which prevents RAC1 activation by RAC-specific guanine nucleotide exchange factors^45^, as a RAC1 inhibitor was evaluated by directly assessing the level of active RAC1 by pull-down with GST-PBD and analyzing the co-precipitated RAC1 (Fig. 6C).

**Figure 7:**
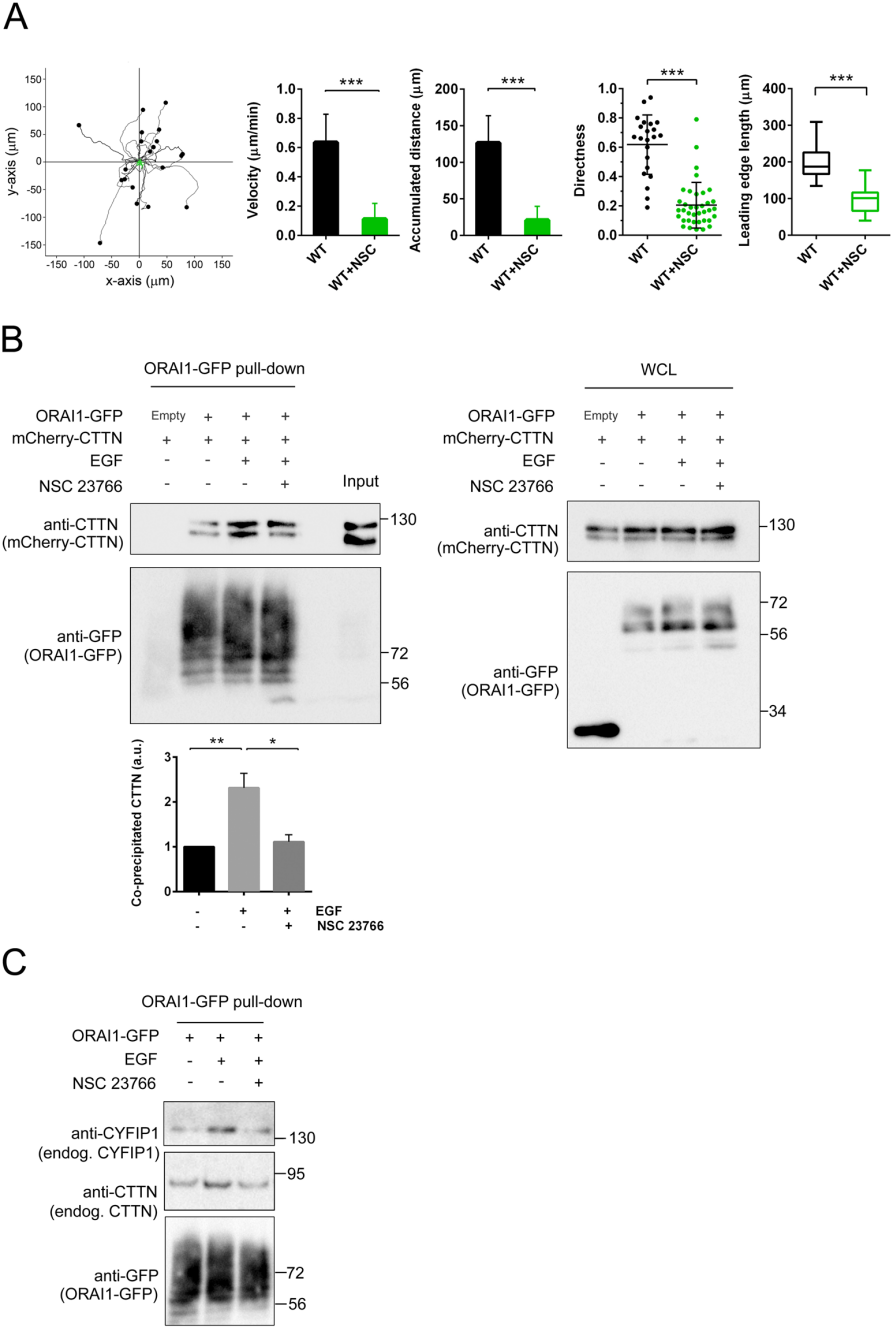
Inhibition of RAC1 impaired cell motility and reduced co-precipitation of ORAI1 with markers of the leading edge. *Panel A*: Tracking of U2OS cells (black symbols) and cells treated with NSC 23766 (green symbols) in a 2D random motility assay. Representative traces per condition are shown (20–21 per condition). The cells were monitored for speed, accumulated distance, and directness. The data in the panels are from 2 independent assays per condition and a minimum of 36 cells per condition. The leading edge length was measured using bright-field images of wild-type live cells growing in collagen-coated culture dishes. When required, cells were incubated with NSC 23766 for 8 h before the analysis. A minimum of 37 cells from 3 independent experiments were measured. *Panel B*: U2OS cells were transfected for the expression of ORAI1-GFP (or the empty vector) and mCherry-CTTN. Cells were starved overnight with FBS-free medium and treated with 50 ng/ml EGF for 3 min. Treatment with NSC 23766 was performed as indicated in the Fig. 6. ORA1-GFP was pulled down, and the co-precipitated mCherry-CTTN analyzed by immunoblot. Total ORAI1-GFP pulled down was assessed with an anti-GFP antibody. The quantification of ORAI1-CTTN co-precipitation was evaluated with an anti-CTTN antibody from 3 independent experiments. Whole cell lysates (WCL) were assessed for the level of mCherry-CTTN and ORAI1-GFP as the experimental control. Blots are representative of 3 independent experiments. *Panel C*: Cells were transfected for the expression of ORAI1-GFP and treated with EGF or NSC 23766 as indicated above. ORAI1-GFP was pulled down and the level of co-precipitated endogenous CYFIP1 and CTTN was analyzed by immunoblot. ORAI1-GFP pulled down was analyzed with an anti-GFP antibody. Blots are representative of 3 independent experiments. Full-length blots are presented in Supplementary Fig. S13.

Because surface ORAI1 was concentrated at the leading edge, where it colocalized with leading edge markers, we analyzed the effect of RAC1 inhibition on lamellipodia formation and cell directness. As shown in Fig. 7A, the treatment of U2OS cells with NSC 23766 reduced cell speed, total distance migrated, cell directness, and the lamellipodia length, similarly to what had been observed in ORAI1-deficient cells (Fig. 1), confirming that RAC1 activity and ORAI1 are essential for the efficient development of the lamellipodia.

As a consequence of the RAC1 inhibition by NSC 23766, the level of ORAI1-CTTN interaction was reduced. This interaction was monitored by pulling down ORAI1-GFP from cells expressing Cherry-CTTN (Fig. 7B), as well as the co-precipitation with endogenous CTTN (Fig. 7C). While EGF stimulated this co-precipitation, the pretreatment of cells with NSC 23766 reduced ORAI1-CTTN co-precipitation, a result that confirmed the role of RAC1 in the translocation of ORAI1 to lamellipodia. As stated above, CYFIP1 is one of the subunits of the WRC. Because CYFIP1 interacts with the active GTP-bound form of RAC1, we also monitored the co-precipitation of ORAI1 with CYFIP1 in response to EGF. As shown in Fig. 7C, ORAI1-CYFIP1 co-precipitation was sensitive to RAC1 inhibition, a result that strongly supported a role for RAC1 in the recruitment of ORAI1 in CTTN- and CYFIP1-rich areas, i.e, the lamellipodia.

**Figure 8:**
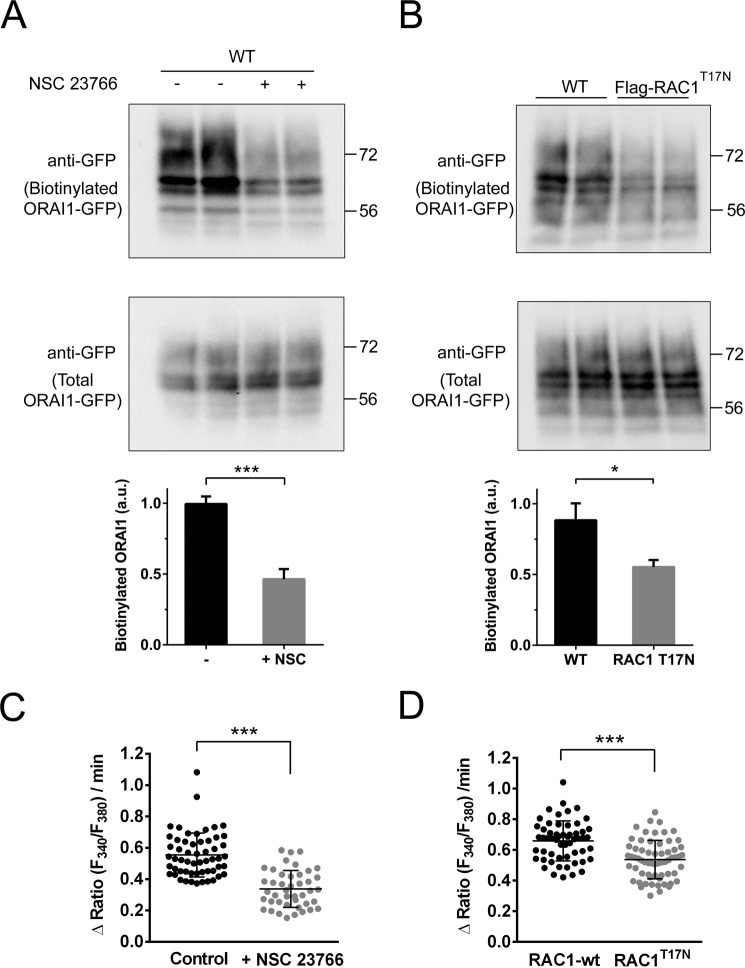
Inhibition of RAC1 reduced surface ORAI1 and SOCE. *Panel A*: U2OS cells were transfected for the expression of ORAI1-GFP and cultured with FBS-containing DMEM. NSC 23766 was added to the culture medium 8 h before the biotinylation assay, when required. Cells were assessed for the level of ORAI1-GFP at the cell surface by a biotinylation assay of proteins at the plasma membrane in live intact cells and subsequent analysis of biotinylated ORAI1-GFP (top panel). Whole cell lysates were analyzed to assess the total amount of ORAI1-GFP (bottom panel). Quantification of results from 2 independent experiments is shown as a bar chart. *Panel B:* Wild-type U2OS cells or cells overexpressing Flag-RAC1^T17N^ were assessed for the level of ORAI1-GFP at the plasma membrane, as in *panel A*. Quantification of results from 2 independent experiments is shown as bar chart. Full-length blots are presented in Supplementary Fig. S13. *Panels C-D*: Cells were loaded with fura-2-AM and treated with 1 μM thapsigargin in Ca^2+^-free Hank’s balanced salt solution. Then, 2 mM CaCl_2_ was added to this assay medium, and the increase rate of the ratio F340/F380 was measured during the first 90 sec after the addition of Ca^2+^. Results from 2 independent experiments are shown as a scatter plot.

In coherence with these results, the treatment of cells with NSC 23766 reduced the amount of ORAI1 at the cell surface (Fig. 8A) as assessed with a biotinylation assay of surface proteins. A similar result was found when the dominant negative mutant RAC1^T17N^ was overexpressed (Fig. 8B) as a strategy to inhibit RAC1. As a control of the specificity of the biotinylation assay for plasma membrane proteins, the absence of biotinylated p38 (cytosolic marker) and the presence of biotinylated Na^+^/K^+^-ATPase (plasma membrane marker) were confirmed by immunoblot (Supplementary Fig. S11). Moreover, the inhibition of RAC1 led to a reduced speed of Ca^2+^ entry in fura 2-loaded cells treated with thapsigargin to deplete intracellular stores (Fig. 8C,D), a result that fits well with the reduced amount of surface ORAI1 when RAC1 is inhibited.

## Discussion

In this study, we have investigated the molecular mechanisms that control ORAI1 localization in migrating cells using the osteosarcoma cell line U2OS, a cellular model with a high invasion and migration potential^46^. We had previously shown that ORAI1 is enriched in CTTN-rich areas^5^, but the regulation of this localization remained unexplained. The results revealed that, upon stimulation with EGF, there is greater co-precipitation of ORAI1 with CTTN, ARP2/3, and CYFIP1, all of which are well-known regulators of cortical cytoskeleton at the leading edge. Active RAC1 (i.e., GTP-bound RAC1) recruits CTTN to the leading edge to help, together with ARP2/3, in the formation of lamellipodia, and CYFIP1 (also known as specifically RAC1-activated protein, or SRA1) is a component of the WAVE regulatory complex^38^. Because we found that the localization of ORAI1 matched that of CTTN, and this latter cytoskeleton regulator is controlled by the activation of RAC1, we studied the hypothesis that ORAI1 may be under the control of RAC1 as well. The inhibition of RAC1 led to a significant reduction of ORAI1 translocation to lamellipodia, to reduced externalization, as well as to reduced co-precipitation with CTTN and CYFIP1 in response to EGF. Thus, our results point to the conclusion that ORAI1 becomes part of the molecular complex that regulates the formation of lamellipodia and that this re-localization of ORAI1 is a RAC1-dependent event.

It is important to note that ORAI1-KO cells showed a significant drop in directional persistence. Because directional persistence positively correlates with lamellipodial persistence^47^, this result supports a significant role for ORAI1 in the control of lamellipodia formation, and suggests that ORAI1 is a necessary member of the set of proteins activated by RAC1 that controls the polymerization and branching of actin at the front of the cell. This conclusion is supported by the sharp drop in the size of the leading edge in ORAI1-KO cells. In this regard, CTTN positively regulates actin branches stability^47^, and the knock-out of CTTN in U2OS cells here strongly reduced lamellipodia size, as well as the cell migration rate and directness (Supplementary Fig. S12), i.e., CTTN-KO cells showed the same features as those found for ORAI1-KO cells.

In addition to ORAI1, other Ca^2+^ channels have been proposed as playing a key role in cell migration. None of them, however, have as yet been described as specific of the leading edge. In contrast, it is known that Ca_V_1.2^48,49^, TRPM7^50^, and TRPV4^51^ are involved in the regulation of the trailing tail contraction. In the first case, Ca^2+^ sparks mediated by Ca_V_1.2 in the rear part of migrating cells act as an essential transducer in establishing the required front-to-rear increasing Ca^2+^ gradient and enhanced directed cell migration^48^. This Ca^2+^ entry at the trailing edge is known to activate a Ca^2+^/calmodulin pathway, mediated by CaMKII, to activate myosin light chain kinase (MLCK) by phosphorylation, and to inhibit MLCK phosphatase, leading to the phosphorylation of actomyosin and promoting the retraction of actomyosin at the trailing edge. In addition, Ca_V_1.2, TRPM7, TRPV4, and other members of the TRPC family are channels with greater conductance than ORAI1 (50–60 pS for TRPV4^52^, 40 pS for TRPM7^53^, 8 pS for Ca_V_1.2^54^, and <1 pS for ORAI1^55^). Thus, the localization of ORAI1 at the leading edge fits well with the proposal that there is an increasing [Ca^2+^]_i_ gradient from the leading edge to the trailing edge, so that [Ca^2+^]_i_ is lower at the leading edge^8,9^.

The fact that Huang *et al*.^56^ found STIM1 at the rear part of the cell appears to contradict the report by Tsai *et al*.^10^, describing a specific enrichment of STIM1 observed at the leading edge and another report of specific enrichment of phospho-STIM1 at the leading edge^5^. In addition, Huang *et al*. found that ORAI1 knock-down or the pharmacological inhibition of this channel did not have any effect on cell polarity, and that ORAI1 did not colocalize with STIM1 in the rear part of the cell^56^. In contrast, our experiments show that deletion of ORAI1 by gene editing decreased polarity and reduced cell directness. Furthermore, the finding that the over-expression of ectopic ORAI1 in KO cells restored the values of directness is evidence for a role of ORAI1 in establishing a front-to-rear axis.

Molecular characterization of Ca^2+^ channels at the leading edge has been difficult to perform. The concept that Ca^2+^ influx at the leading edge is required for membrane ruffling is not new because it had been described in macrophages^6^. This study demonstrated that the inhibition of extracellular Ca^2+^ influx leads to disassembly of F-actin and cessation of ruffling, although it did not elucidate the molecular pathway for this Ca^2+^ influx. Later, our own group reported that the knock-out of ORAI1 reduces membrane ruffling^5^, and that over-expressing ectopic ORAI1 in ORAI1-KO cells led to recovery of the wild-type phenotype.

In this report, we have shown that there is a specific enrichment of ORAI1 at the leading edge in response to EGF, and that the suppression of its expression leads to significant defects in lamellipodia formation and directional persistence of migrating cells.

In support of this role for ORAI1 at the leading edge, STIM1 enrichment has also been found at the front of migrating human umbilical vein endothelial cells^10^, and there is a high level of phospho-STIM1 enrichment at the leading edge^5^ where the highest activities of receptor tyrosine kinase and ERK1/2 have been described^10^. It is important to note that the pool of STIM1 phosphorylated by ERK1/2 binds ORAI1 to activate SOCE^29,32^, and that this phosphorylation releases STIM1 from EB1, a microtubule plus-end binding protein. Since the leading edge is a microtubule-free sheet, this specific localization of ORAI1 and phospho-STIM1 at the leading edge is evidence for a role of SOCE at the front of migrating cells.

Finally, the function of ORAI1 as an effector of RAC1 that has been shown here is particularly important since RAC1 drives tumor initiation. Therefore, targeting specific RAC1 interactors or effectors, such as ORAI1, is a plausible therapeutic strategy that requires detailed knowledge.

## Materials and Methods

### Chemicals

Flp-In T-REx U2OS cells were kindly provided by Dr. Gopal Sapkota (University of Dundee). Cells were tested for contamination before the experiments presented in this report were carried out. Type-I collagen, EGF, and brefeldin A were purchased from Sigma-Aldrich (St. Louis, MO, USA), NSC 23766 from Tocris (Abingdon, UK), 1,1′-dioctadecyl-3,3,3′,3′-tetramethylindocarbocyanine perchlorate (DiI) from ThermoFisher Scientific (Waltham, MA), polyethylenimine from Polysciences, Inc. (Eppelheim, Germany), and Clarity^TM^ and Clarity Max^TM^ ECL blotting substrates from Bio-Rad (Hercules, CA, USA).

### DNA constructs

The construct for expressing mCherry-cortactin was provided by Christien Merrifield and distributed by Addgene (plasmid #27676)^57^. The construct for the expression of human ORAI1-GFP (NM_032790.3) was described and validated elsewhere^5^. The construct for the stable expression of untagged human ORAI1 by retroviral infection was made by amplifying ORAI1 cDNA by PCR to insert *Bam*HI + *Not*I flanking sites. The resulting PCR product was then inserted into *Bam*HI + *Not*I sites of the pBABED-Hygro vector (construct DU37127 from the University of Dundee). The constructs for the expression of Flag-RAC1, Flag-RAC1^T17N^, or Flag-RAC1^G12V^ (constructs DU17293, DU17294, DU17295) can be requested on the website (https://mrcppureagents.dundee.ac.uk/). The plasmids pcDNA3-EGFP-RAC1(wt) and pcDNA3-EGFP-RAC1^Q61L^ were a gift from Klaus Hahn (Addgene plasmids #13719 and #13720)^58^. The plasmid pmCherry-RAC1^G12V^ was a kind gift from Dr. Francesc Tebar (University of Barcelona, Spain). The plasmid CSCW-Gluc-YFP for the stable expression of the *Gaussia* luciferase was a kind gift from Dr. Bakhos A. Tannous (Harvard Medical School, USA).

DNA constructs used for transfection were purified from *E. coli* DH5α using ZymoPure kits in accordance with the manufacturer’s protocol. All DNA constructs were verified by DNA sequencing at the DNA Sequencing Service of the University of Dundee (www.dnaseq.co.uk), or at the Sequencing Facility of STAB, University of Extremadura. Transfection of cells with DNA constructs was performed with 1 μg plasmid DNA per 10-cm dish and polyethylenimine in serum-containing medium, 24–30 h prior to the beginning of the experiments.

### Antibodies

Mouse monoclonal anti-cortactin (clone 4F11), rabbit polyclonal anti-CYFIP1 antibody, mouse monoclonal anti-ARP2/3 complex antibody (clone 13C9), and rabbit polyclonal anti-TGN46 were purchased from Merck Millipore (Darmstadt, Germany), mouse monoclonal anti-RAC1 and rabbit polyclonal anti-Sec13a were from ThermoFisher Scientific (Waltham, MA, USA), and the antibodies against phosphorylated ERK1/2 (Thr202/Tyr204), total-ERK1/2, total-PAK1, phospho-PAK1/2 (Thr423/Thr402), total p38 MAPK, and the anti-GFP antibody from Cell Signaling Technology Inc. (Danvers, MA, USA). The antibodies against EEA1 and LAMP1 were from BD Biosciences (San Jose, CA, USA). The polyclonal antibody for detecting GM130 was from R&D systems. GFP-Trap resin was purchased from Chromotek GmbH (Martinsried, Germany), the anti-Na^+^/K^+^-ATPase and the anti-GAPDH antibodies were from Santa Cruz Biotechnologies (Dallas, TX, USA). All secondary HRP- and Alexa Fluor-labeled antibodies were from ThermoFisher Scientific.

### Culture of cells

U2OS cells were cultured in DMEM with 10% (v/v) FBS, 2 mM L-glutamine, 100 U/ml penicillin, and 100 μg/ml streptomycin, in a humidified atmosphere of air/CO_2_ at 37 °C. Cells under stimulation with EGF were starved for 8–10 h in serum-free and phenol red-free RPMI 1640 medium, and then stimulated with 50 ng/ml EGF in the same culture medium. Resting cells were starved for the same time in phenol red-free RPMI medium. In all cases, cell culture dishes and glass coverslips were treated with type-I collagen solution (0.1 mg/ml) for a minimum of 2 h at 37 °C. The treatment with NSC 23766 was performed by preincubation of cells with 50 μM of the inhibitor for 8–10 h.

### Generation of stable cell lines

Flp-In T-REx U2OS cells able to inducibly express Flag-RAC1, Flag-RAC1^G12V^, or Flag-RAC1^T17N^ were generated as reported previously^32^. Stably transfected cells (resistant to hygromycin and blasticidin) were cultured in Dulbecco’s modified Eagle’s medium (DMEM) with 10% (v/v) foetal bovine serum, 2 mM L-glutamine, 100 U/ml penicillin, 100 μg/ml streptomycin, 100 μg/ml hygromycin B, and 15 μg/ml blasticidin in a humidified atmosphere of air/CO_2_ at 37 °C. Cells were treated with 1 μg/ml doxycycline for 20–24 hours before assays to induce expression of tagged RAC1.

To stably transfect ORAI1-KO cells with a mammalian expression vector containing the ORAI1 cDNA, retroviral transduction was performed as described previously^59^. Briefly, Phoenix amphotropic retroviral packaging cells were transfected (6 μg DNA per 10-cm dish) with the pBABED-Hygro-ORAI1 plasmid. At 24 and 48 h post-transfection, ORAI1-KO U2OS cells were incubated with the virus-containing medium with 4 μg/ml polybrene. The culture was extended 48 h, and hygromycin selection (0.5 mg/ml) was performed for 6 days.

### Generation of genetically modified cells using CRISPR/Cas9 genome editing

The method to generate ORAI1-KO U2OS cells by CRISPR/Cas9 genome editing has been described previously^5^. ORAI1-KO cells were characterized for the lack of ORAI1 expression and lack of store-operated Ca^2+^ entry^5^.

To generate a CTTN-KO cell line, we analyzed the *CTTN* locus using both NCBI and Ensembl (NC_000011.10, ENSG00000085733) and found three verified transcriptional variants (NM_005231, NM_138565, and NM_001184740). A paired nickase approach was adopted to minimise off-targeting and exon 3 was chosen as the optimal region for CRISPR targeting, being the farthest upstream common coding exon large enough to harbour suitable paired guides. The guide pair (sense 5′-gATGACGCGGGGGCCGATGAC(TGG) and antisense 5′- gCCTGGGCGATGGACACAGCG(TGG)) was subsequently identified using the Sanger Institute CRISPR webtool (http://www.sanger.ac.uk/htgt/wge/find_crisprs) and chosen on the basis of having a low combined off-targeting score. Complementary oligos with *Bbs*I compatible overhangs were designed according to the Zhang method^60^, and these dsDNA guide inserts ligated into *Bbs*I-digested target vectors; the antisense guide was cloned into the spCas9 D10A expressing vector pX335 (Addgene Plasmid #42335) and the sense guide into the puromycin selectable plasmid pBABED P U6 (University of Dundee). Cells were co-transfected with 1 μg of each plasmid using polyethylenimine in a 10-cm dish. Following 24 h of recovery and a further 48 h of puromycin selection (2 μg/ml), the cell pool was single-cell sorted by FACS, and clones were cultured and analyzed for CTTN protein depletion by immunoblotting and by DNA sequencing. Genomic DNA was isolated, and the region surrounding the target site of the guide RNAs amplified by PCR (forward primer: 5′-CTCGGTAGTTTTGGCCAAAGGG; reverse primer: 5′-AAACCCAGGAGAGCCACTTCC). The resulting PCR products were subcloned using the StrataClone Blunt PCR Cloning Kit (Agilent Technologies), and twelve bacterial colonies picked and sequenced to verify indels. Sequencing of the exon 3-PCR fragments from the CTTN-KO cells revealed a 20 + 23 base-pair deletion, confirming the successful KO of the *CTTN* locus.

DNA constructs for the generation of CTTN-KO (constructs DU52284, DU52303) can be requested on our reagents website (https://mrcppureagents.dundee.ac.uk/).

### Immunoblots and pull-down

Cells were lysed in the following buffer: 50 mM Tris-HCl (pH 7.5), 1 mM EGTA, 1 mM EDTA, 1% (w/v) Nonidet P40, 1 mM sodium orthovanadate, 50 mM NaF, 5 mM Na_3_PO_4_, 0.27 M sucrose, 1 mM dithiothreitol (DTT), 1 mM benzamidine, and 0.1 mM phenylmethylsulfonyl fluoride. After lysis with 0.75-1 ml of ice-cold lysis buffer/10 cm-dish, samples were clarified by centrifugation at 20000 g for 20 min at 4 °C. Then the samples were sonicated with five 10-sec pulses using a Branson Digital Sonifier with a setting of 45% amplitude. Protein concentration was determined using the Coomassie Protein Assay Reagent (ThermoFisher Scientific).

For immunoblots, lysates (10–40 μg) were subjected to electrophoresis on polyacrylamide gels and subsequent electroblotting to nitrocellulose membranes. Membranes were blocked for 1 h at room temperature (RT) in blocking buffer: TBS-T (Tris-buffered saline buffer, pH 7.5, with 0.2% Tween-20) containing 10% (w/v) non-fat milk. Then the membranes were incubated overnight at 4 °C with the primary antibody diluted in TBS-T containing 5% BSA (for antibodies from Cell Signaling Technology) or 10% (w/v) non-fat milk. The primary antibody concentration was as follows: 1 μg/ml for anti-CTTN, anti-CYFIP1, and anti-GFP antibody; 1:3000 dilution for the anti-GAPDH antibody; 1:2000 dilution for the anti-Na^+^/K^+^-ATPase antibody; 1:1000 dilution for anti-total-ERK1/2, anti-phospho-ERK1/2, anti-total-PAK1, anti-phospho-PAK1/2, and anti-RAC1 (from the RAC1 pull-down and detection kit); and 1:500 dilution for anti-ARP2/3 complex (clone 13C9) and anti-p38 MAPK. Membranes were washed with TBS-T, and then incubated with anti-mouse, anti-rabbit, or anti-sheep IgG horseradish peroxidase (HRP)-conjugated secondary antibody (1:10 000 dilution) for 1 h at RT in blocking solution. In all cases, chemiluminescence substrate was added to the membranes and the signal recorded with the ChemiDoc XRS+ imager (BioRad). The recorded signal was quantified by volumetric integration using Image Lab software.

ORAI1-GFP was purified with GFP-Trap agarose beads. Beads (5 μl) were added to clarified cell lysates (0.8–1 mg), followed by incubation for 1 h at 4 °C with gentle shaking. The GFP-Trap beads were then washed once with 1 ml lysis buffer containing 0.15 M NaCl and twice with 50 mM Tris–HCl, 0.1 mM EGTA, pH 7.5. Proteins were eluted from the beads by the addition of 15 μl NuPAGE-LDS sample buffer to the beads. The eluted proteins were reduced by the addition of 10 mM DTT followed by heating at 72 °C for 10 min, and samples were subjected to electrophoresis on 6.5% polyacrylamide gels^5^.

### Immunolocalization

Immunolocalization of endogenous EEA1, GM130, LAMP1, Sec13a, and TGN46 was performed on cells fixed with 4% paraformaldehyde in PBS for 10 min at room temperature. Permeabilization was performed with 0.2% Triton X-100 and blocking with 3% fish skin gelatin in PBS + 0.2% Tween-20 for 30 min at room temperature. Cells were incubated overnight at 4 °C with the antibodies at the following dilutions: EEA1 (1:100), GM130 (1:100), LAMP1 (1:50), Sec13a (1:50), and TGN46 (1:500). Anti-mouse, rabbit, or sheep IgG labeled with Alexa Fluor 594 were used as a secondary antibody (diluted 1:500, 20 min at RT). Coverslips were mounted onto glass slides with Hydromount (National Diagnostics). Images of fixed cells were taken on a Nikon Ti-E inverted epifluorescence microscope with a Plan Apochromat 100× (NA 1.45) oil immersion objective.

### Wide-field epifluorescence microscopy

GFP- and mCherry-tagged proteins were imaged in 4% paraformaldehyde-fixed cells. The excitation settings were: 480/30 nm excitation filter, 535/40 nm emission filter, and 505 nm dichroic mirror for GFP, and 562/40 nm excitation filter, 641/75 nm emission filter, and 593 nm dichroic mirror for mCherry (Semrock). Images of fixed cells were taken on a Nikon Ti-E inverted epifluorescence microscope with a Plan Apochromat 100× (NA 1.45) oil immersion objective. In all cases, cells were z-scanned (0.2 μm-step sections), and image deconvolution was performed with 20 iterations and the 3D Landweber deconvolution method (NIS-AR software).

### Cell surface protein biotinylation assays

ORAI1 externalization was assessed with the Cell Surface Protein Isolation kit (Pierce, ThermoFisher Scientific), following the manufacturer’s instructions. Briefly, ORAI1-GFP expressing cells grown on 10 cm-diameter culture dishes were treated with or without NSC 23766, washed with PBS, and incubated with EZ-LINK sulfo-NHS-SS-biotin for 30 min at 4 °C. The labelling reaction was stopped by the addition of the quenching solution, and cells were lysed using the lysis buffer provided by the manufacturer, containing Halt protease inhibitor cocktail. Protein concentration was calculated, and an aliquot of the lysate was saved for immunoblotting analysis of total ORAI1-GFP. The biotinylated ORAI1-GFP was isolated by incubation of 0.5 ml of cell lysate (0.3 mg total protein) with NeutrAvidin agarose gel, eluted with sample buffer containing DTT, and assessed by immunoblot with an anti-GFP antibody.

### RAC1 activity assay

To detect active (GTP-bound) RAC1 we used the active RAC1 pull-down and detection kit (ThermoFisher Scientific). Briefly, U2OS cells were grown on 10 cm-diameter culture dishes, and after 2 days they were starved overnight in serum-free medium. Cells were then treated with 50 ng/ml EGF for 0.5 min-5 min with or without NSC 23766, and then lysed using the cell lysis buffer provided by the manufacturer. After determining the protein concentration with the compatible 660 nm protein assay (Pierce, ThermoFisher Scientific), an aliquot of the lysate was saved for the quantification of the total RAC1 by immunoblot. In parallel, 0.7 ml of the cell lysate (0.6 mg total protein) was incubated at 4 °C with gentle rocking for 1 h together with 20 μg of GST-human PAK1-PBD protein in a column containing GSH resin. This resin was washed, and proteins eluted with loading buffer. Proteins were separated on 4–12% SDS-PAGE followed by blotting to a nitrocellulose membrane. Total RAC1 and active RAC1 were detected using the monoclonal anti-RAC1 antibody provided.

### *Gaussia* luciferase secretion assay

U2OS cells were transduced with the construct CSCW-Gluc-YFP, as described elsewhere^44^. Cells expressing *Gaussia* luciferase (Gluc) were cultured in 96-well plates at a 10^5^ cells/well density. After 6 h, expression of Flag-RAC1^T17N^ was induced with 1 μg/ml doxycycline for 22 h. In parallel, cells expressing endogenous RAC1 only were treated with 50 μM NSC 23766 for the last 8 h of culture before measuring luciferase activity. Luciferase activity in the culture medium (secreted Gluc) and the intracellular luciferase activity were evaluated with the *Gaussia* Luciferase Glow Assay Kit (ThermoFisher Scientific) following the instructions of the manufacturer. Luminescence was measured using a Varioskan Flash luminometer (ThermoFisher Scientific). Controls were done by incubating cells with 5 μg/ml brefeldin A for 2–4 h.

### Motility and cell migration assays

For random cell migration assessment, cells were plated on 0.1 mg/ml collagen-coated 35 mm-dishes at 40–50 × 10^3^ cells/dish in 2 ml bicarbonate-free Leibovitz’s L-15 medium supplemented with 10% FBS. The cells were left to adhere for 90 min before performing the experiments. Cells were time-lapse recorded on a Nikon Ti-E microscope equipped with a UNO-Okolab stage incubator (37 °C) using a 10× bright field objective, at a frame rate of 1 every 2 min, and a minimum duration of 6 h. Manual cell tracking was performed by using Tracking Tool^TM^ Pro (Gradientech, Uppsala, Sweden). Speed, total distance, and directness (Euclidean distance/total distance) during 200 min were calculated with the migration tracks of >36 cells per condition from 3 independent experiments.

### Cytosolic free calcium concentration measurement

[Ca^2+^]_i_ was measured in fura-2-AM-loaded cells as described elsewhere^5,13,29,32,59,61–63^. Excitation fluorescence wavelengths were selected with 340/26 and 387/11 nm filters (Semrock), and emission fluorescence with a 510/10 nm filter. All measurements were performed at 36–37 °C. Excitation/emission conditions were controlled by the NIS-Elements AR software. Depletion of Ca^2+^ stores was triggered by incubating cells with 1 μM thapsigargin (Tg) in Ca^2+^-free HBSS with the following composition: 138 mM NaCl; 5.3 mM KCl; 0.34 mM Na_2_HPO_4_; 0.44 mM KH_2_PO_4_; 4.17 mM NaHCO_3_; 4 mM Mg^2+^(pH = 7.4). SOCE was measured by monitoring the increase of the [Ca^2+^]_i_ after the addition of 2 mM CaCl_2_ to the Tg-containing medium.

### *In vivo* invasion assay

The experiments complied with the Guidelines of the European Union Council (Directive 2010/63/EU) and the Spanish RD 53/2013. Experiments and procedures were performed as approved by the Bioethical Committees of the University of Murcia (approval references #75/2014, #216/2014, and #395/2017). *In vivo* invasion assay was performed as reported previously^64^. Briefly, U2OS cells were washed with PBS and labeled with 4 ng/μl DiI (ThermoFisher Scientific) in PBS + 5% FBS. The cells were incubated sequentially at 37 °C for 15 min and 4 °C for 15 min and washed by centrifugation with 67% PBS + 5% FBS. Finally, cells were resuspended in 67% PBS + 5% FBS. Two hundred cells per embryo were then injected in the yolk sac of transparent *roy*^*a9/a9*^; *nacre*^*w2/w2*^ (casper) zebrafish larvae^65^ of 48 h post-fertilization (hpf) and after 5 days at 35 °C the larvae were analyzed for human U2OS cells dissemination by fluorescence microscopy (see experimental design in Supplementary Fig. S5).

U2OS cell invasion score was calculated as the percentage of zebrafish U2OS cell-invaded larvae over the total number of larvae analyzed taking into account also the number of tumor foci per larvae. Three tumor foci were established to score a larva as positive for invasion. Furthermore, larvae positive for invasion were also distinguished in three groups considering the number of positive foci per larvae: 3–5 foci per larvae, 5–15 foci per larvae and >15 foci per larvae. Images were acquired using a Leica MZ16F fluorescence stereo microscope and processed using ImageJ software (http://rsb.info.nih.gov/ij/).

### Image analysis

Persistence and protrusion distance were calculated as described elsewhere^66^. Basically, cells were grown on collagen-coated culture dishes. After a minimum of 4 h after plating, cells were selected when they showed free migration without cell-cell contacts for 2 h. Kymographs were prepared by plotting a 3 pixel-width line to evaluate lamellipodial persistence and protrusion.

Circularity was evaluated by measuring the perimeter and total area of individual cells. Circularity was defined as 4 x *pi* x area/perimeter.

Evaluation of Pearson correlation coefficient (Fig. 2D) was performed by designing circular ROIs of 1.1–1.18 μm^2^ over the cellular periphery. The number of ROIs per cell was 70–90 in order to evaluate all the perimeter of the cell that remained free of cell-cell contacts.

Normalization of cortical ORAI1/CTTN over total ORAI1/CTTN was performed by defining a single ROI for every cell that enclosed the cortical region, excluding cell-cell contact sites. An additional ROI surrounding the entire cell was designed to evaluate total fluorescence. Fluorescence intensity for GFP- and mCherry- channels was recorded in both ROIs, and data of cortical ORAI1 (or CTTN)/total ORAI1 (or CTTN) was calculated and plotted (see Supplementary Fig. S7).

The NIS-Elements AR software package was used to assess all features.

### Statistical analysis of data

Statistical analyses were done using the unpaired *t*-test. Differences between groups of data were taken statistically significant for *p* < 0.05. The *p*-values are represented as follows: (n.s.) *p* > 0.05, (*) *p* < 0.05, (**) *p* < 0.01, and (***) *p* < 0.001.

## Supplementary information

Supplementary Information

Supplementary Movie 1

Supplementary Movie 3

Supplementary Movie 8

Supplementary Movie 9

Supplementary Movie 10
