# Supplementary material for: RAC1-Dependent ORAI1 Translocation to the Leading Edge Supports Lamellipodia Formation and Directional Persistence
Source: Sci Rep. 2020 Apr 20;10:6580. doi: 10.1038/s41598-020-63353-5 (PMC7171199; doi:10.1038/s41598-020-63353-5)
Supplement: Supplementary file 1 — Supplementary Information [file 41598_2020_63353_MOESM1_ESM.pdf]

Supplementary information:

RAC1-DEPENDENT ORAI1 TRANSLOCATION TO THE LEADING EDGE SUPPORTS LAMELLIPODIA FORMATION AND DIRECTIONAL PERSISTENCE

Aida M. Lopez-Guerrero[1,*], Noelia Espinosa-Bermejo[1,*], Irene Sanchez-Lopez[1], Thomas Macartney[2], Carlos Pascual-Caro[1], Yolanda Orantos-Aguilera[1], Lola Rodriguez-Ruiz[3], Ana B. Perez-Oliva[3], Victoriano Mulero[3], Eulalia Pozo-Guisado[4], Francisco Javier Martin-Romero[1]

Supporting data for Figure 1:

Movies S1 and S2. Wild-type U2OS cells (Movie S1) and ORAI1-KO cells (Movie S2) were monitored for speed, accumulated distance, and directness in a 2D random motility assay. Cells (40 000-50 000 per dish) were plated onto collagen-coated 35-mm dishes and monitoring of cell motility was started at least 90 min after plating to ensure appropriate adhesion. Movies are time-lapses of 132 frames recorded at a rate of 1 frame every 2 min.

Supporting data for Figure 4:

Movies S8, S9, and S10. Full time-lapse sequences of the experiments shown in the Figure 4. U2OS cells stably expressing Flag-RAC1 (wild-type) (movie S8), Flag-RAC1^{G12V} (movie S9) or Flag-RAC1^{T17N} (movie S10) were transfected for the transient expression of ORAI1-GFP and mCherry-CTTN. Emission of fluorescence was recorded every 3 sec for 4 min.

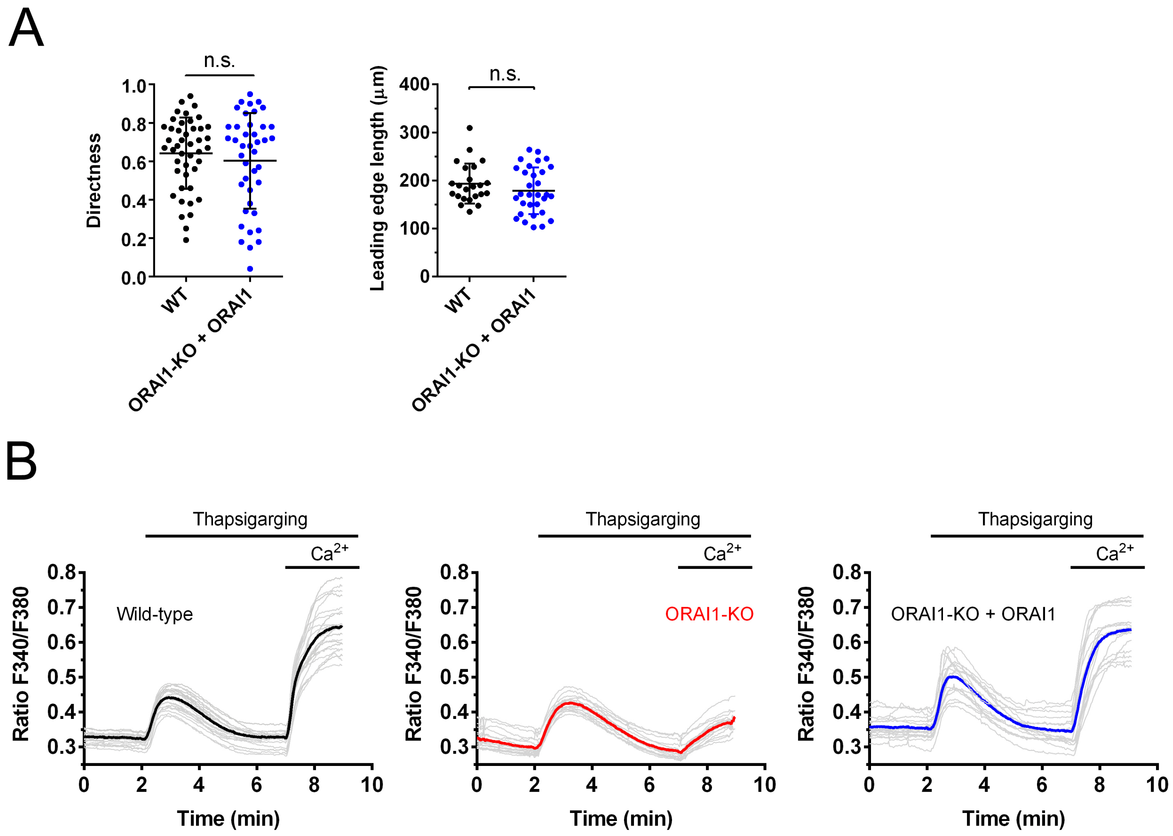

Figure S3. Panel A: ORAI1-KO cells stably transduced for the over-expression of ORAI1 were monitored for directness in a 2D random motility assay. Data from 3 independent assays and >40 cells are shown dot plot. Note that data from WT cells have been plotted in the Fig. 1.A and 1.B to facilitate comparison with data from ORAI1-KO cells. Panel B: Store-operated Ca(2+) entry was assessed in ORAI1-KO cells stably expressing ectopic untagged ORAI1 to evaluate the functional recovery of a wild-type phenotype. The analysis was performed by triggering store emptying with 1 µM thapsigargin in fura-2-loaded cells incubated in a Ca(2+)-free medium, and then adding 2 mM Ca(2+) to the assay medium. Plots are traces from a single experiment, representative of 3 independent experiments per experimental condition.

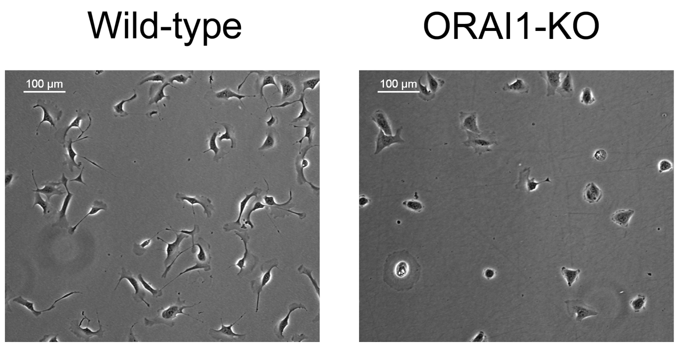

Figure S4. Representative images supporting the experiment to measure the leading edge length in wild-type and ORAI1-KO cells. Images are full-frame photographs of non-fixed wild-type and ORAI1-KO cells growing on collagen-coated dishes. Recordings were performed with the NIS-AR software under phase contrast microscopy using a Plan Achromat 10× Ph1 Nikon objective.

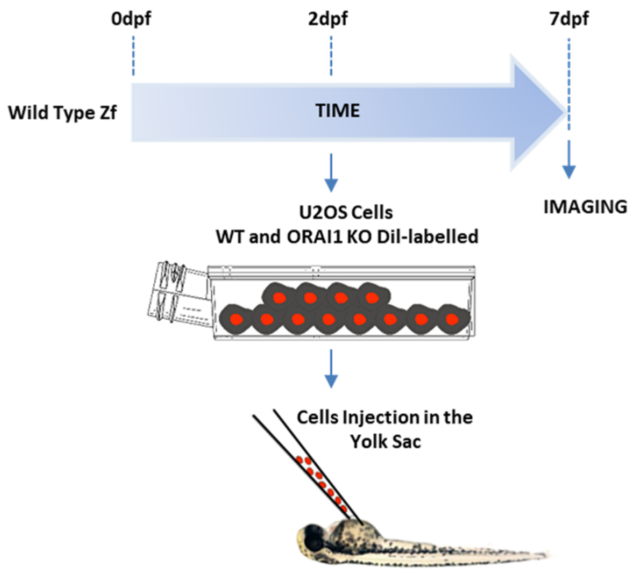

Figure S5. Experimental design of the in vivo invasion assay. U2OS cells were labelled with 1,1'-dioctadecyl-3,3,3',3'-tetramethylindocarbocyanine perchlorate (DiI, ThermoFisher Scientific), in a buffer containing 5% FBS in PBS. Two hundred cells/embryo were then injected in the yolk sac of casper zebrafish larvae 48 h post-fertilization (hpf) and, after 5 days at 35°C, cell dissemination in the larvae was examined by fluorescence microscopy. A cell invasion score was calculated as the percentage of larvae invaded by human U2OS cells relative to the total number of larvae analyzed.

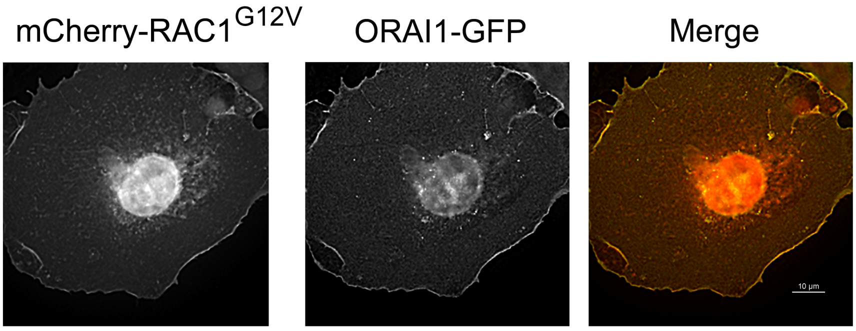

Figure S6. U2OS cells were transfected for the expression of mCherry-RAC1^{G12V} and ORAI1-GFP. Twenty-four hours after transfection, cells were fixed and visualized under wide-field epifluorescence microscopy. Cherry and GFP channels were recorded sequentially using independent filter blocks. Images are representative of 22 cells from 2 independent experiments.

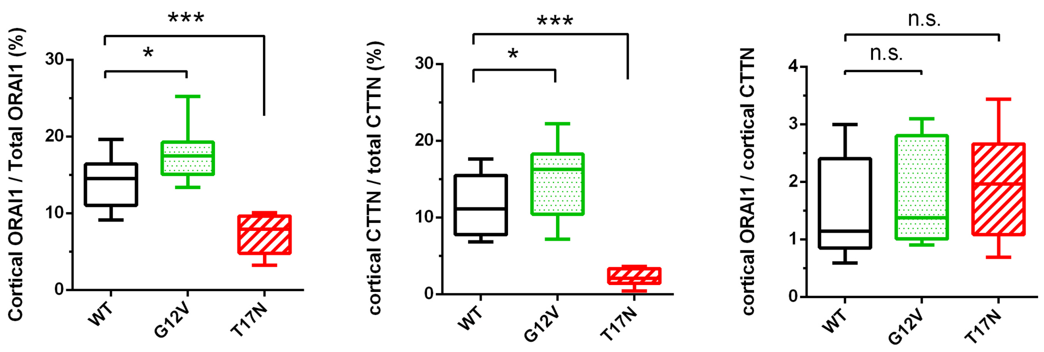

Figure S7. Quantification of cortical and total ORAI1 and CTTN in cells studied in Figure 3 (panel B). Individual cells (n > 25 for every experimental condition) were analyzed by measuring total intensity in GFP and mCherry channels with the NIS-Elements AR software. Cortical fluorescence intensity was analyzed from a single region of interest (ROI) covering the entire periphery of the cell, excluding areas of cell-cell contact. ROIs were typically 0.66 ± 0.22 µm-width. Total fluorescence intensity was analyzed with a single ROI that included the whole individual cell.

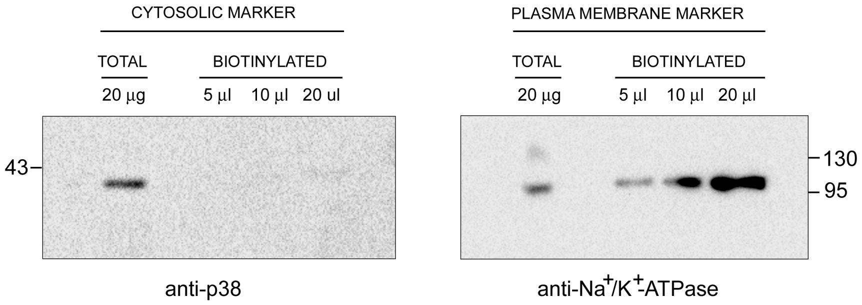

Figure S11. U2OS cells were transfected for the expression of ORAI1-GFP to assess the level of biotinylated ORAI1. As a control to test the efficiency of the assay, 20 µg of total lysate, or increasing volumes of biotinylated samples eluted from the columns were assessed to study the level of p38 MAPK, as a cytosolic marker, and for the Na^+/K^+-ATPase, as a plasma membrane marker. The lack of biotinylated p38 and the enrichment of Na^+/K^+-ATPase in the biotinylated fraction confirmed the specificity of this assay.

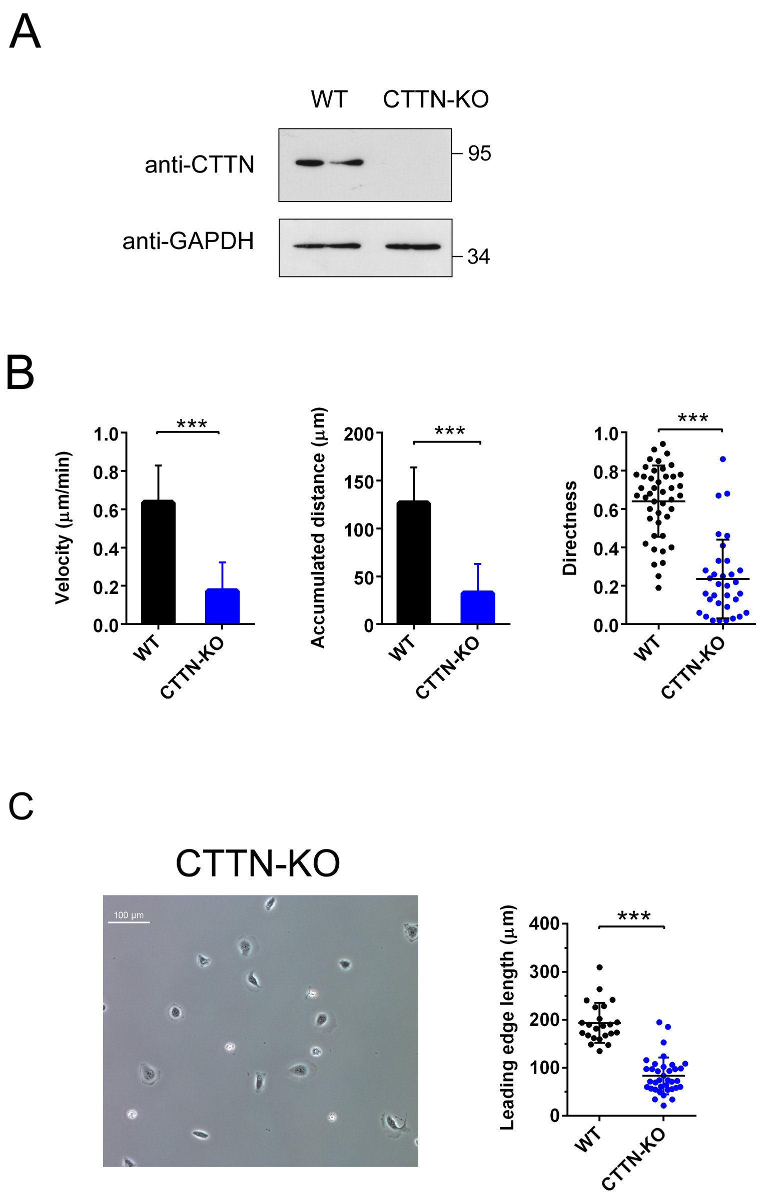

Figure S12. U2OS cells were edited with CRISPR/Cas9 as detailed in Methods to generate a U2OS cell line deficient for the expression of cortactin (CTTN-KO). Panel A: Immunoblot to detect endogenous cortactin (CTTN) from whole cell lysates obtained from the parental cell line (WT) and CTTN-KO cells. Panel B: Wild-type U2OS cells (black symbols) and CTTN-KO cells (blue symbols) were monitored for speed, accumulated distance, and directness in a 2D random motility assay. Data from 3 independent assays per condition (n = 44 WT cells; n = 33 KO cells) are shown as a bar chart or dot plot. Note that data from WT cells have been plotted in the Fig. 1.A to facilitate comparison with data from ORAI1-KO cells. Panel C: The leading edge length was measured using bright-field images of wild-type cells (n = 23) and CTTN-KO cells (n = 36) from 4 independent experiments. Bar = 100 μm. Note that data from WT cells have been plotted in the Fig. 1.B to facilitate comparison with data from ORAI1-KO cells.

UNCROPPED BLOTS

Fig 2.A

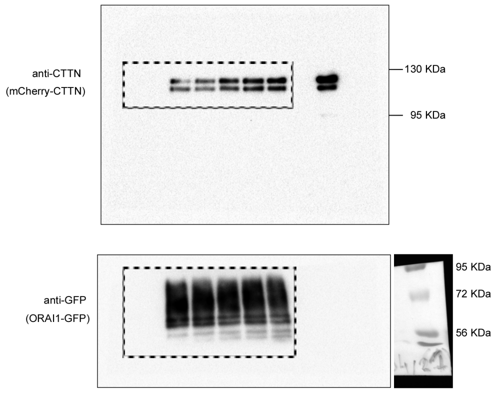

Fig 2.B

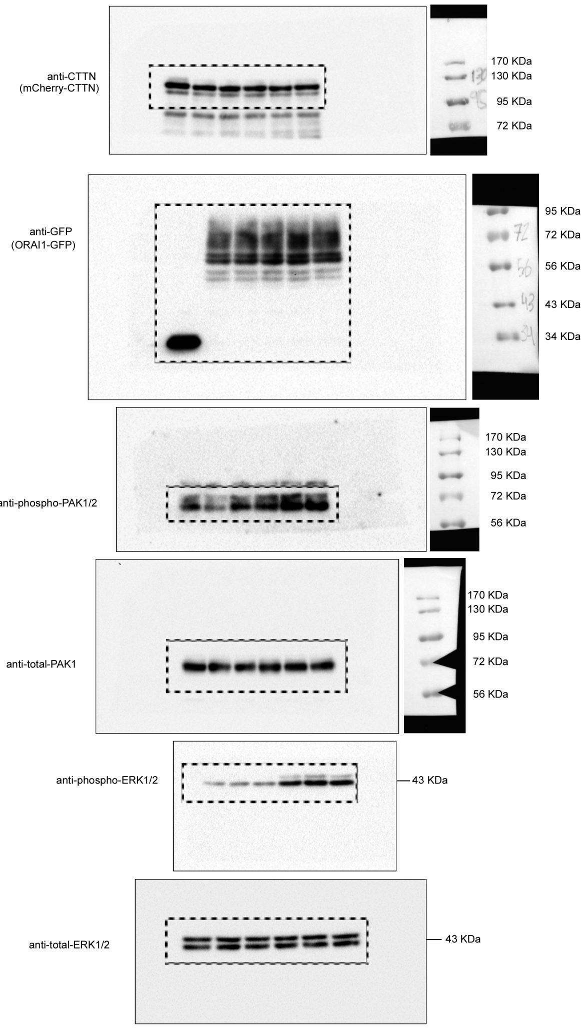

Fig 2.C

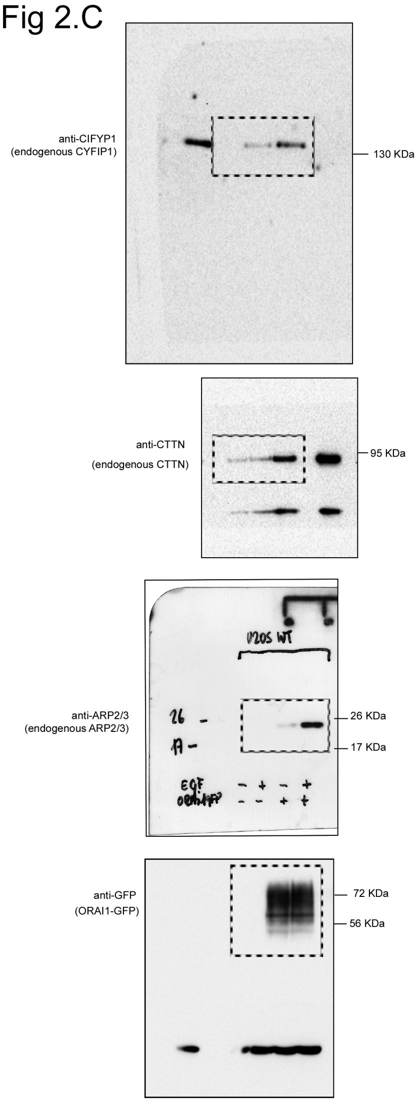

Fig 5.C

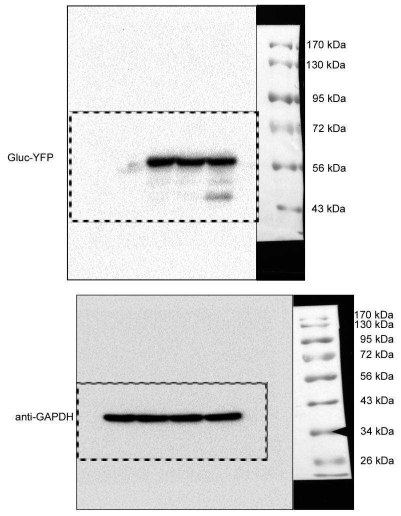

Figure S13. Uncropped full-length pictures of western blotting membranes presented in the main Figs. 2, 5, 6, 7, 8, and Supplementary Figs. S11 and S12. Membranes were often cut to enable blotting for multiple antibodies.

UNCROPPED BLOTS

Fig. 6.A-C

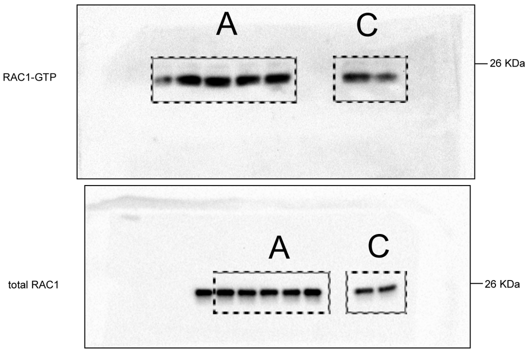

Fig. 6.B

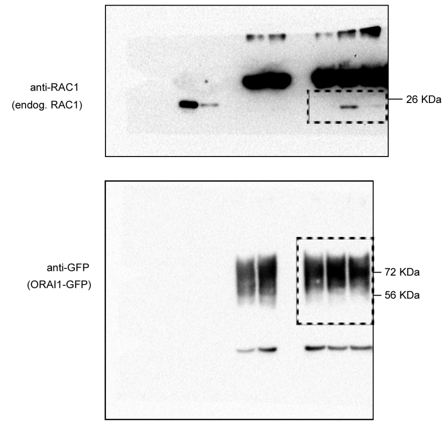

Fig. 7.B

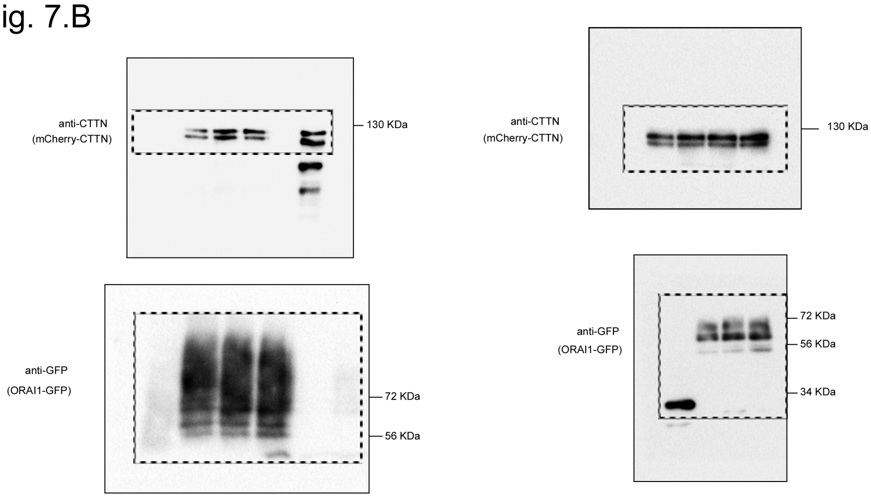

Fig. 7.C

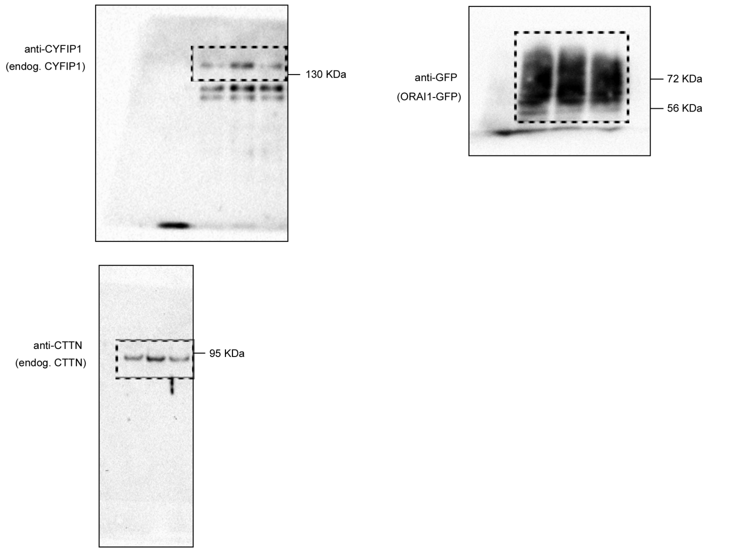

Figure S13. Uncropped full-length pictures of western blotting membranes presented in the main Figs. 2, 5, 6, 7, 8, and Supplementary Figs. S11 and S12. Membranes were often cut to enable blotting for multiple antibodies.

UNCROPPED BLOTS

Fig. 8.A

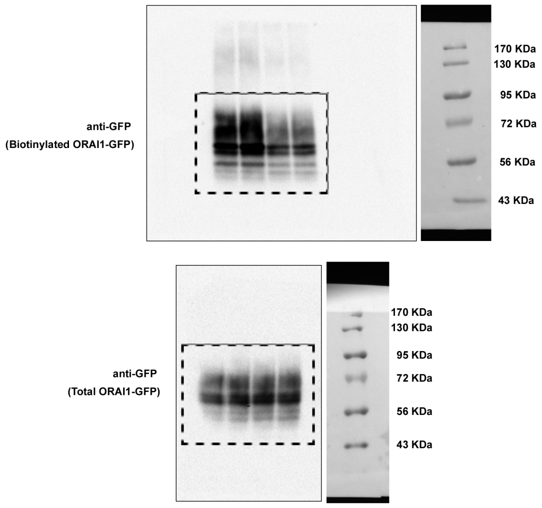

Fig. 8.B

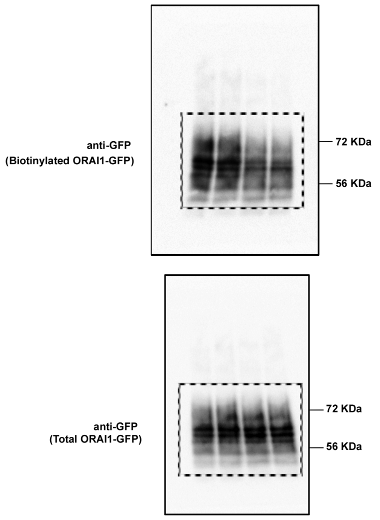

Fig. S11

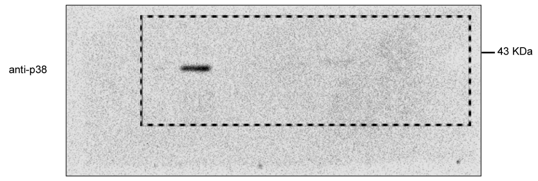

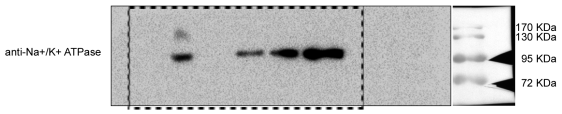

Fig. S12.A

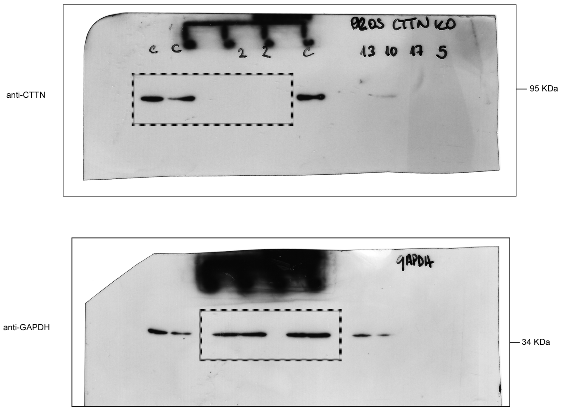

Figure S13. Uncropped full-length pictures of western blotting membranes presented in the main Figs. 2, 5, 6, 7, 8, and Supplementary Figs. S11 and S12. Membranes were often cut to enable blotting for multiple antibodies.
